# Conformational Dynamics and Stability of Bilayers Formed by Mycolic Acids from the *Mycobacterium tuberculosis* Outer Membrane

**DOI:** 10.3390/molecules28031347

**Published:** 2023-01-31

**Authors:** Liana A. Savintseva, Ilya S. Steshin, Alexander A. Avdoshin, Sergey V. Panteleev, Alexey V. Rozhkov, Ekaterina A. Shirokova, Grigory D. Livshits, Alexander V. Vasyankin, Eugene V. Radchenko, Stanislav K. Ignatov, Vladimir A. Palyulin

**Affiliations:** 1 Department of Chemistry, Lobachevsky State University of Nizhny Novgorod, 603022 Nizhny Novgorod, Russia; 2 Department of Chemistry, Lomonosov Moscow State University, 119991 Moscow, Russia

**Keywords:** mycolic acids, mycobacterium, cell wall, tuberculosis, outer membrane, conformational dynamics, molecular dynamics

## Abstract

Bilayers of mycolic acids (MAs) form the outer membrane of *Mycobacterium tuberculosis* that has high strength and extremely low permeability for external molecules (including antibiotics). For the first time, we were able to study them using the all-atom long-term molecular dynamic simulations (from 300 ns up to 1.2 μs) in order to investigate the conformational changes and most favorable structures of the mycobacterial membranes. The structure and properties of the membranes are crucially dependent on the initial packing of the α-mycolic acid (AMA) molecules, as well as on the presence of the secondary membrane components, keto- and methoxy mycolic acids (KMAs and MMAs). In the case of AMA-based membranes, the most labile conformation is W while other types of conformations (sU as well as sZ, eU, and eZ) are much more stable. In the multicomponent membranes, the presence of the KMA and MMA components (in the W conformation) additionally stabilizes both the W and eU conformations of AMA. The membrane in which AMA prevails in the eU conformation is much thicker and, at the same time, much denser. Such a packing of the MA molecules promotes the formation of a significantly stronger outer mycobacterial membrane that should be much more resistant to the threatening external factors.

## 1. Introduction

Today, the emergence and spread of drug-resistant infections creates one of the major challenges for the modern medicine. If left unchecked, it could bring the Humanity back to a time when antibiotics were unknown, threatening with huge mortality from the diseases that, it would seem, have long been defeated. Among such infections, perhaps the most dangerous is tuberculosis (TB) [1], a serious chronic bacterial infectious disease caused by the pathogenic *Mycobacterium tuberculosis* (*Mtb*) mycobacteria [2,3]. According to the World Health Organization (WHO), TB is one of the most widespread and socially significant infections: despite it being a preventable and curable disease, every year, about 10.6 million people develop tuberculosis and 1.6 million people die worldwide, making it the leading cause of death from a single infectious agent [4]. Although the disease is usually localized in the respiratory organs, it may also involve other organs and tissues. In fact, one of the characteristic features of tuberculosis is the variety of its clinical and pathomorphological manifestations as well as broad adaptability to changing and/or unfavorable environmental conditions (including host organism) and external physical, chemical, and pharmaceutical factors [5].

It is commonly accepted that one of the key causes for this resiliency is the *Mycobacterium tuberculosis* cell envelope [6,7]. Atypical among bacteria, it has an elaborate dense multilayered structure, devoid of the majority of transporters, that helps to protect it against the actions of the host immune cells, adverse environments, and many antimicrobial agents. It is thus not surprising that various inhibitors of specific processes of the cell wall biosynthesis [8,9,10,11] have long been established as a major group of antitubercular drugs. Moreover, the approaches aiming to directly target and modulate the mycobacterial membranes are currently seen as very promising [12]. Among the cell envelope layers, perhaps the most important protective role belongs to the wax-like outer membrane [7,8,13] that has high strength and extremely low permeability for external molecules (especially the relatively hydrophilic ones, including some antibiotics [6]). 

**Figure 1:**
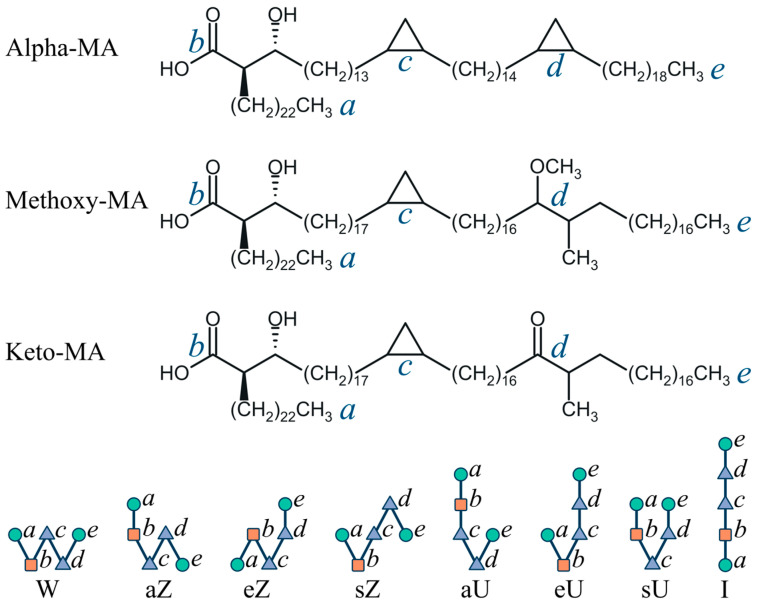
Mycolic acid types in *Mtb* and designations of their possible conformations.

Although some structural and compositional variations exist between the different species and strains [14,15], overall, the outer membrane (OM), or mycomembrane, of *Mycobacterium tuberculosis* and related species and genera consists of the molecules of mycolic acids (MAs), i.e., 2-alkyl-3-hydroxy long-chain fatty acids, and their derivatives that are arranged into a bilayer membrane [7,8,13]. In particular, α-mycolic acids (AMAs), methoxymycolic acids (MMAs), and ketomycolic acids (KMAs) are normally found in the OM (Figure 1). All of them are β-hydroxy acids with linear chains of 60–90 carbon atoms [14]. These molecules have a linear 24-carbon aliphatic chain at the α-position with respect to the COOH group and a hydroxy group at the β-position that correspond to the (*2R,3R*) stereochemistry (fragment *a–b* in Figure 1). Two more groups are found at positions *c* and *d* inside a long hydrocarbon chain. AMA has *cis*-cyclopropane rings inserted in both the proximal (*c*) and the distal (*d*) positions with respect to the carboxy group. It is the most common type of MAs in *Mtb*, usually comprising from 50% to 70% of the total MA content [16,17,18]. MMA and KMA, collectively comprising from 30% to 50% of the total MA content, have either *cis*- or α-methyl-*trans*-cyclopropane rings inserted in the proximal (*c*) position, and, respectively, methoxy and methyl or keto and methyl substituents in the distal (*d*) position [13,14]. In some cases, the properties of various MAs and the effects of their stereochemistry have been elucidated using chemically synthesized panels of compounds [15].

In addition to differences in the functional groups, long MA molecules can adopt different conformations due to the bending of their molecular chains. The most commonly used classification of the MA conformations is based on the mutual arrangement of the points *a*, *b*, *c*, *d*, *e* located at the ends of the chain and in the positions of the functional groups. These conformations are commonly referred to as W, aZ, sZ, eZ, aU, sU, eU, and I (Straight) [19,20] (Figure 1), with the overall assignment scheme called the WUZ classification. Most of the MA molecules in the inner leaflet of the outer membrane are covalently bound to the terminal arabinofuranosyl units of arabinogalactan (AG) polysaccharide that, together with peptidoglycan, forms the insoluble *Mtb* cell wall skeleton [10,13,14,21]. The outer leaflet contains MAs in the form of their trehalose esters such as dimycolates (TDM) and monomycolates (TMM), as well as free mycolates and various long-chain lipids of different classes [13]. 

Despite the general understanding of the outer membrane structure, a number of important issues remain unexplored, such as the conformational composition of mycolic acids that determines the way molecules are packed in the membrane. Experimental methods do not allow us to elucidate it because of the poor ability of the membrane substance to crystallize, and theoretical methods have so far been limited to studying isolated conformations of molecules in solution and vacuum or rather rough coarse-grained modeling. At the same time, the packing of molecules in a membrane is one of the most important factors that determine the thickness of membranes, their density, the ability to withstand the action of external chemical agents and temperature, as well as the permeability to antibiotics and other small molecules. An alternative approach to the permeability prediction based on the machine learning structure–property models [22] is also useful but the model applicability domain can be limited by the available data.

Based on the indirect evidence, such as the experimentally determined thickness of the outer membrane (7–8 nm) and high MA packing density in the OM, it was suggested that the mycolic acids of the outer mycobacterial membrane should be in the W-shaped conformation, because long MAs simply would not fit in the membrane [23,24]. At the same time, the X-ray diffraction studies have shown that mycolic acids are oriented parallel to each other and perpendicular to the cell wall plane [25]. The importance of molecular dynamics studies of the hydrocarbon tail packing in the bulk of the membrane is emphasized due to the impossibility of its experimental determination [23].

AMAs change the W shape quite easily, adopting various elongated conformations depending on the ambient pressure and temperature [18]. Their elasticity is equivalent to that of liquid condensed films of common fatty acids such as stearic acid (100–250 mN/m), and the values are more or less the same for all α-MAs. It has also been experimentally established and confirmed by theoretical research that the presence of an α-methyl-*trans*-cyclopropane group stabilizes the W packing for KMA and MMA [26]. 

The all-atom molecular dynamics simulations were used to determine the conformational diversity of MAs in vacuum and various solvents [20] and the AMA+AG complexes in water [27]. The observation concerning the tendency of KMA and MMA to fold over their α-methyl-trans-cyclopropane group was confirmed. MAs exhibit similar folding in vacuum and water, with most of the folded conformations distributed around the W conformation, although the molecules are more flexible in vacuum than in water. However, the behavior of molecules in a membrane can be completely different. 

It was shown that, when using the MARTINI coarse-grained force field to model a monolayer separating water and vacuum, AMAs do not fold into the W conformation, but form a wide variety of the U conformations [19]. When the monolayer was compressed, the MA chains approached each other, adopting a narrower U shape, and an ordered monolayer was formed (followed by its eventual destruction). However, in a bilayer, the situation may be different because the leaflets of the outer membrane would constrain each other, which may not allow the W conformation to unfold so freely.

In the present work, for the first time, we perform a molecular dynamics study of the evolution of bilayer membranes of mycolic acids in water using the all-atom force fields. We assume that the bilayer model of the outer membrane based on mycolic acids is much closer to reality than the single-layer one. We consider a wide range of membranes formed by three main representatives (AMA C_78_H_152_O_3_, KMA C_87_H_170_O_4_, MMA C_85_H_168_O_4_) both in their individual single-component compositions and in the mixed compositions close to the experimentally determined composition of the cell wall. For each membrane composition, we study the conformational dynamics, including the kinetic data on the interconversions of various conformations and their relative stability, as well as analyze the membrane thickness and profile. The main goal of this work is to establish the most stable conformations and packing methods of mycolic acids in bilayer structures and to study the influence of the conformational composition on the membrane thickness and density, eventually aiming to better understand the structure and properties of the outer membrane of the *Mtb* cell wall as well as to find the ways to influence it when developing novel antibacterial agents. 

## 2. Results and Discussion

### 2.1. General Modeling Assumptions

As noted above, the goal of this study was to explore the molecular dynamics and analyze the evolution of bilayer membranes of mycolic acids in water using the all-atom force fields. We assume that the bilayer model of the outer membrane is much closer to reality than the single-layer ones previously employed in the literature, with the non-polar regions interfacing vacuum or a non-polar organic solvent. As a first approximation, we decided to limit ourselves to symmetrical bilayers with essentially identical leaflets comprising the unmodified (but possibly mixed) MA molecules. In this way, we were able to focus on the key general patterns of their behavior while avoiding the extra degrees of freedom (and additional uncertainty) associated with the covalent linkages to arabinogalactan in the inner leaflet and to trehalose residues in the outer leaflet (which could also contain non-MA lipids). 

Nevertheless, the free (unmodified) MA molecules were modeled in their un-ionized (neutral) form even though one could expect their *pK_a_* values to be close to 4–4.5, implying virtually complete ionization at the physiological pH. However, the neutral carboxylic group would be more representative of the ester groups present in the covalently linked MA molecules. Moreover, in the actual *Mtb* infection or culture, the remaining free acid groups are exposed to a gel-like microenvironment rich in ions and polar molecules that can reduce their dissociation compared to the bulk solvent.

### 2.2. Dynamic Conformational Diversity in Single-Component Membranes

**Figure 2:**
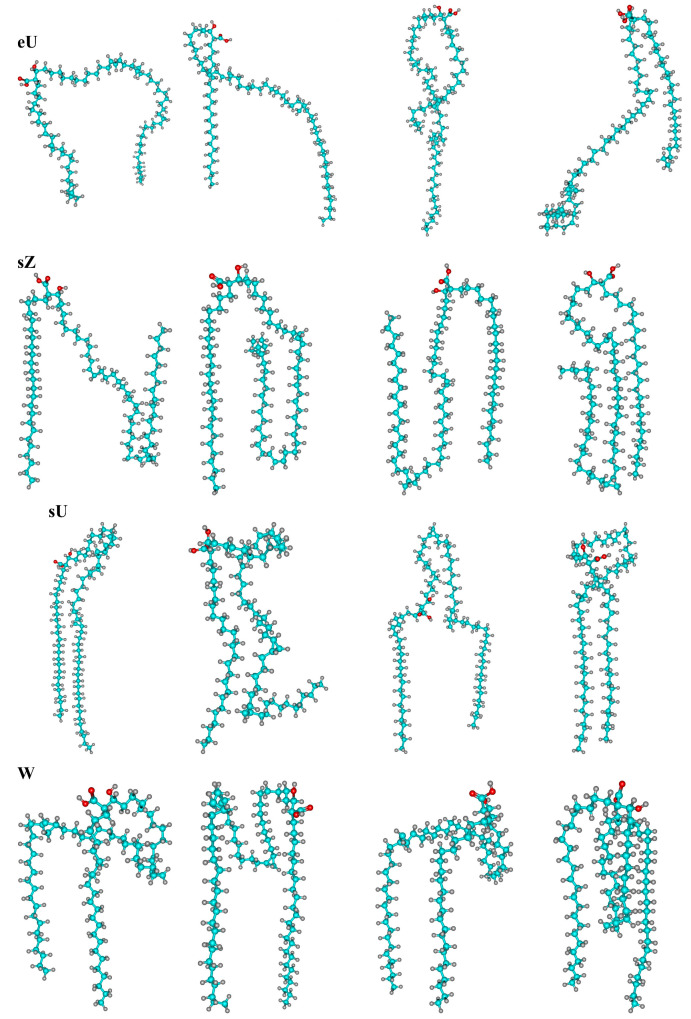
Typical structures of the most abundant conformation classes eU, sZ, sU, and W formed in the single-component AMA-W membrane during 300 ns MD.

For AMA, the initial membrane models for molecular dynamics simulations were constructed from the W, sZ, and eU conformations. In the case of the W conformation, the initial shape of the molecules changed significantly in the course of molecular dynamic evolution for 300 ns, turning into other types of conformations. Even when the same type was formally retained, the molecules often were significantly distorted due to the bending of various aliphatic fragments and the changes in dihedral angles, which are not taken into account by the WUZ classification. Therefore, although many molecules formally belong to one of the seven WUZ classes, the specific shape of the molecules could significantly differ from the standard one for the class. Particularly frequent are bends close to the *b–c* fragment, leading to the formation of “loops” near the COOH group. In many cases, these loops deviate from the original direction of the molecule and tend to occupy a position along the membrane surface. The greatest conformational diversity is achieved in the case of W-type conformations, which quickly give rise to a large number of new structures. Figure 2 shows typical representatives of the structures formed during the evolution in this system for *t* = 300 ns. In contrast to the W structure, the other two types of single-component membranes studied (sZ and eU) turn out to be more stable, and the product conformations of various types that accumulate over 300 ns usually amount to about 10%. 

In most cases, there are no clear differences in energy between the structures formed in the AMA-W system and no noticeable energy barriers between them if they are considered in vacuum or in solution. However, in the membranes of the sZ or eU type, unlike W, the MA molecules retain their shape for a long time, which allows us to speak of the “membrane conformers”. Such a definition is close to the concept of “packing”, although it is not as strict as packing in a molecular crystal. 

### 2.3. Influence of Composition and Initial Packing of Membranes on Gyration Radii of Molecules

To analyze the membrane structure, it is interesting to evaluate the continuous structural parameters that characterize the compactness of the membrane components or their various conformations. Among these parameters, the most indicative are the mass-weighted gyration radii $R_{g}$:

$$R_{g}={\left[\frac{\sum_{i}m_{i}{\left(r_{i}-r_{CM}\right)}^{2}}{\sum_{i}m_{i}}\right]}^{1/2}$$

where $m_{i}$ and $r_{i}$ are the masses and coordinates of atoms, $r_{CM}$ are the coordinates of the center of mass, and the summation is carried out over all atoms in the molecule.

**Figure 3:**
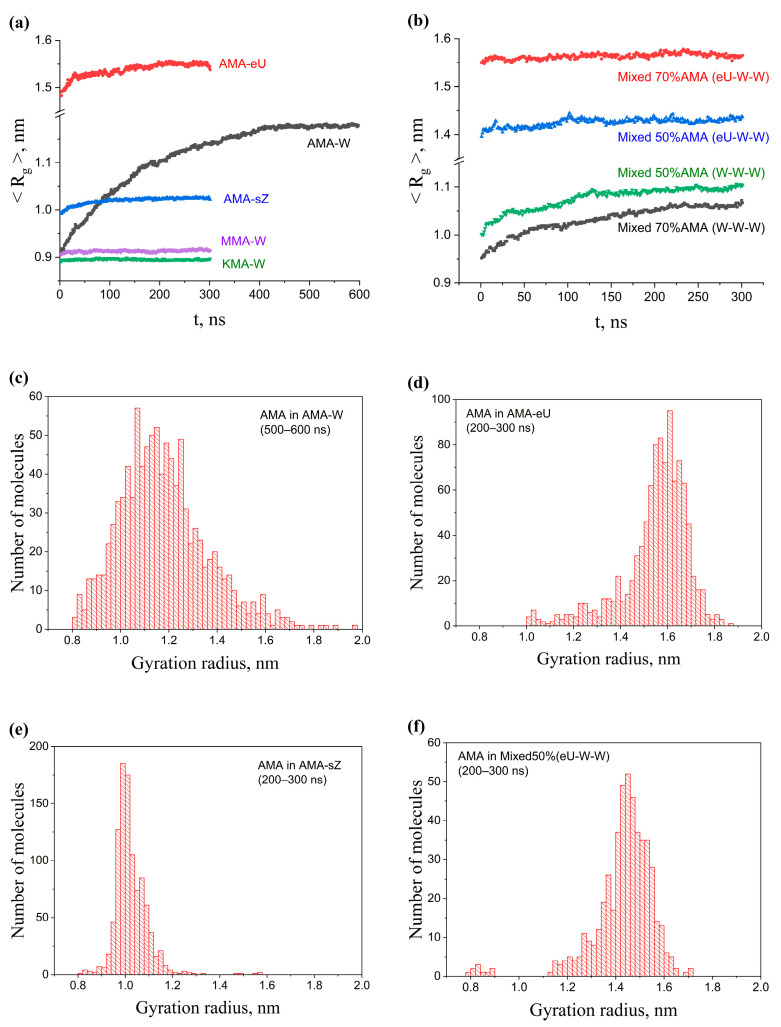
Time evolution of the average mass-weighted gyration radius of 1000 AMA molecules: (**a**) in the single-component membranes of different initial construction and composition (AMA-W, AMA-eU, AMA-sZ, KMA-W, and MMA-W); (**b**) in the multicomponent membranes of different initial construction and composition (140W–30W–30W, 140eU–30W–30W, and 100eU–50W–50W); (**c**–**f**) Distribution of $R_{g}$ for 1000 AMA molecules in membranes with different composition and structure at the time interval of 200–300 ns of NPT MD.

The strongest dependence of the $R_{g}$ values on the evolution of the system for the molecules in a single-component membrane was observed for AMA molecules in the initial W conformation (Figure 3a). The gyration radius was changing for more than 300 ns, and the $R_{g}$ value increased by more than 25% relative to the initial one. This indicates that the W conformation, even in the membrane, was very unstable and mobile. Noticeable changes in the membrane structure come to an end after about 400 ns. Membranes with the original sZ and eU structures were much more stable, and the single-component membranes based on KMA and MMA were even more stable. In the process of MD evolution, changes in their gyration radii were almost imperceptible. It should be noted that the $R_{g}$ values of the single-component membrane molecules were stabilized at different levels (with the exception of MMA and KMA). This means that the structure of the membrane strongly depends on the way the molecules were initially packed, and membranes with different structures can retain them for a long time.

The addition of the KMA and MMA components in a 30% amount had almost no effect on the $R_{g}$ value of AMA (i.e., the “folding” of these molecules) in the membrane based on AMA-eU; however, this value was significantly reduced when KMA and MMA were added in the amount of 50% (Figure 3b). At the same time, even a 30% addition of KMA/MMA significantly reduced the gyration radius (i.e., made AMA molecules more compact) in the case of a multicomponent membrane based on AMA-W. 

The difference in the gyration radii did not arise from random deviation factors when averaging similar structures. The distributions of $R_{g}$ differed significantly for the W, sZ, and sU structures in the single-component membranes (Figure 3c–e), and the shapes of these distributions only slightly changed after 200 ns. In the case of multicomponent membranes (Figure 3f), the distribution of $R_{g}$ for AMA molecules was noticeably shifted to lower values, almost without changing the shape of the distribution. This confirms the conclusions made for these membranes based on the average $R_{g}$ values. 

In addition to $R_{g}$, other characteristics were also used to quantitatively describe the compactness of molecules, including the massless gyration radii $r_{g}$ and the hydrodynamic radii $R_{hyd}$. The unweighted (massless) gyration radius is defined as:

$$r_{g}={\left(\lambda_{1}+\lambda_{2}+\lambda_{3}\right)}^{1/2}$$

It is calculated on the basis of eigenvalues $\lambda_{k}\;\left(k=1,2,3\right)$ of massless gyration tensors, taking into account the coordinates only of the carbon atoms in a linear polymer chain [28]:

$$T_{\alpha \beta }=\frac{1}{2N^{2}}\underset{i}{\sum }\underset{j}{\sum }\left(r_{i}^{\alpha }-r_{j}^{\alpha }\right)\left(r_{i}^{\beta }-r_{j}^{\beta }\right)$$

where $r_{i,j}^{\alpha ,\beta }$ are the components of the coordinate vectors of atoms *i* and *j* (*α*, *β* = *x*, *y*, *z*), *N* is the number of atoms. The hydrodynamic radius $R_{hyd}$ [29] is a parameter that describes the characteristic size of a molecule when moving in a viscous medium:

$$R_{hyd}={\left[\frac{1}{N^{2}}\underset{i>j}{\sum }{\left|r_{i}-r_{j}\right|}^{-1}\right]}^{-1}$$

The time dependences of the average $r_{g}$ and $R_{hyd}$ values of MAs in the membranes of various compositions turn out to be completely similar to the dependence of $R_{g}$, and in the case of $r_{g}$ their values differ from $R_{g}$ by only 5–10%, while the $R_{hyd}$ values are significantly higher. Because $r_{g}$ and $R_{hyd}$ are important parameters of polymeric structures in a condensed state that are frequently used for the description of viscosity and other mechanical properties of (bio)polymers, we show the time dependences of the $r_{g}$ and $R_{hyd}$ values in the Supplementary Materials (Figure S1).

### 2.4. Distribution of Molecular Shape Parameters

Calculation of the gyration tensors of molecules makes it possible to represent any instantaneous MA structure as the so-called characteristic ellipsoid of gyration (also referred to as ellipsoid of inertia) with semiaxes $a,b,c=\sqrt{\lambda_{k}}$, where $\lambda_{k}\;\left(k=1,\;2,\;3\right)$ are the eigenvalues of the gyration tensor. The elongation or oblateness of the ellipsoids of molecules affect the thickness and density of the entire membrane. The elongation of the molecules can be described by the dimensionless quantity of prolateness P [28]:

$$P=\frac{\left(2a-b-c\right)\left(2b-c-a\right)\left(2c-a-b\right)}{2{\left(a^{2}+b^{2}+c^{2}-ab-bc-ac\right)}^{3/2}}$$

That takes values from –1 (an infinitely thin disk) to +1 (an infinitely thin rod).

**Figure 4:**
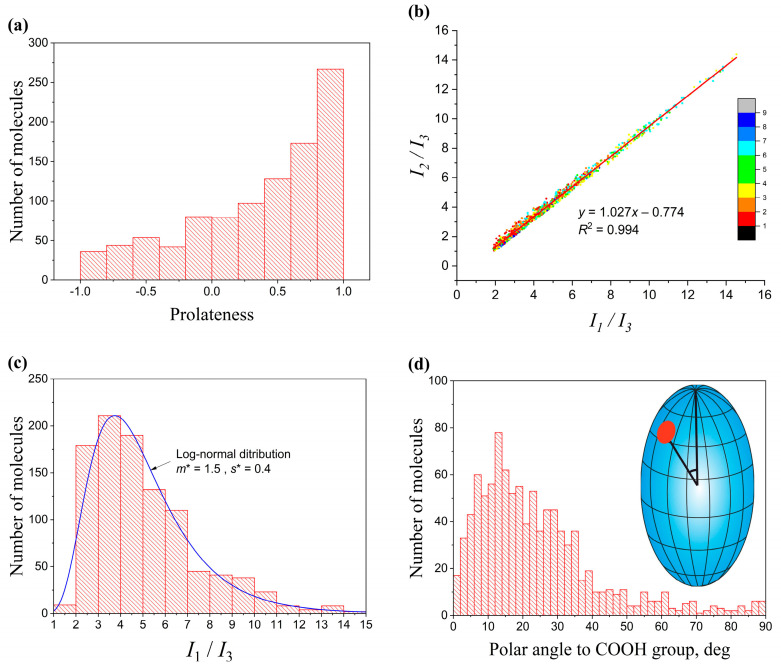
(**a**) Distribution of prolateness P for 1000 AMA molecules in a membrane with initial structure W over the 200–300 ns MD interval; (**b**) Relationship of ratios $I_{1}/I_{3}$ and $I_{2}/I_{3}$ for a sample of 1000 structures from 200 to 300 ns MD. The solid line is the bisector $I_{1}=I_{2}$ (ellipsoid of revolution); (**c**) The distribution of the ratio of maximum and minimum moments of inertia follows a log-normal distribution ($m^{*}$ and $s^{*}$ are the log-normal mean and standard deviation); (**d**) Representation of an MA molecule as an ellipsoid with the position of the COOH group (red circle) and the histogram of the polar angles of the COOH group on the ellipsoid of inertia.

As is seen from the distribution of P values (Figure 4a), although some of the molecules had P<0 (oblate ellipsoids), most of the molecules tended to P>0, and many of them had P values close to one. Additionally, the magnitude of the asymmetry of ellipsoids can be characterized by comparing the principal moments of inertia of the molecules. For most MA molecules in the membrane, the relations $I_{1}\sim I_{2}>I_{3}$ take place, which correspond to a prolate ellipsoid. The ratios of the moments of inertia $I_{1}/I_{3}$ and $I_{2}/I_{3}$ are almost equal to each other and do not depend on the value of $I_{3}$ and the type of conformation (Figure 4b). This means that ellipsoids are ellipsoids of revolution, regardless of the type of conformation. This fact is rather unusual since the conformations themselves (Figure 2) are very different in their structure. Another interesting fact is that the distribution of the ratio of the larger moment to the smaller one $I_{1}/I_{3}$ (i.e., the prolateness of the ellipsoids) is described with high accuracy by a log-normal distribution (Figure 4c). The log-normal distributions are widely found in nature. The fundamental reason for the appearance of such a distribution in this case could be that a change in the size of the ellipsoid leads to an increase in the energy of its rotational or librational motion within the membrane. The number of molecules with a given energy is described by the Boltzmann distribution. If the average energies of the molecules are normally distributed, then the number of molecules is the logarithm of the energy and is distributed log-normally.

An additional structural parameter of the molecules in the membrane is the position of the hydrophilic groups of the molecule (primarily COOH) on the surface of its ellipsoid of inertia, which can be measured by the polar angle that this group forms with the major semiaxis of the ellipsoid. It is easy to see that in most molecules the COOH groups deviate from the major axis of the ellipsoid, which explains the characteristic tilt of MA molecules in the membrane by 10–20° that is observed in most membranes (Figure 4d).

### 2.5. Kinetics of Conformation Changes in Membranes

#### 2.5.1. Single-Component AMA Membranes

**Figure 5:**
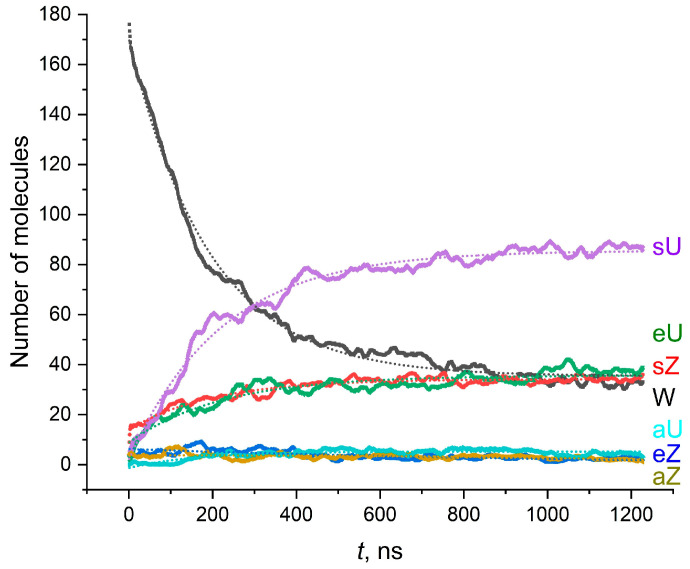
Changes in conformational composition in the bilayer AMA-W membrane during 1200 ns NPT MD (*T* = 300 K). Dotted lines are the fitted exponential regression models.

In order to quantitatively describe the long-term kinetic changes within the MA membranes and estimate the time interval needed for the formation of a pseudo-equilibrium membrane (the membrane where the conformational composition does not change over longer time scales), we carried out a long (1200 ns) MD run for the AMA-W membrane. During the MD production run, the membranes composed of the W conformations undergo fast transformation to other conformation types (Figure 5). Among these new conformations, the most abundant are the sZ, sU, and eU shapes. One can conclude that the W conformation of AMA is structurally quite unstable even within the dense membranes. It is transformed to other conformations up to almost 50% during 300 ns. The time dependence of the W shape concentration decrease (and the corresponding accumulation of other conformations) is described well by the mono-exponential regression model:

$$N\left(t\right)=N_{\infty }+A\exp\left(-t/\tau \right)$$

The characteristic time of conformational transformations was typically in the range of 150–250 ns, which corresponds to the monomolecular transformation rate of about 54.5–86.5 μs^–1^ (Table 1). It can also be seen that the accumulation of sU structures has a rather remarkable induction period of about 100 ns. The exponential concentration decay corresponds to the first order reaction kinetics (monomolecular reaction), i.e., to the reaction rate dependent only on the concentration of a single conformation. However, a closer look at the changes in the conformational composition (Figure 5) reveals more subtle kinetic features. Namely, in the region of 0–150 ns, the decrease in the partial concentration of the W form is closer to a linear law than to an exponential one, while the exponential dependence begins to manifest itself to a greater extent after 150 ns. The linear change in the number of W conformations corresponds to the zero-order reaction kinetics. It is typical for the reactions limited by external factors, for example, the number of external photons or the number of active centers on the surface. We can assume that this linear kinetics is a consequence of the limitation of the energy absorbed by the system due to the fact that the temperature control algorithm does not allow for the rapid absorption/release of the thermal energy required for the conversion of a large number of molecules.

In general, the analysis of the data (Table 1 and Figure 5) shows that the most favorable conversion product of W is sU, whereas eU and sZ are present in slightly smaller and approximately equal amounts. The number of the aZ, eZ, and aU structures is negligible. This is in agreement with the findings from the previous studies [19] that the W structure of AMA is unstable and undergoes rapid changes. The equilibrium number of the molecules, extrapolated to infinite evolution time, is also highest in the case of the sU conformation while the difference between the sZ, eU, and W conformations is small. It is necessary to take into account the approximate nature of assigning molecular structures to a limited number of standard conformations. In fact, the molecules adopt much more diverse conformations which cannot be accurately described by the WUZ classification, and their assignments are strongly dependent of the classification method (e.g., the method of clustering used for the Straight structures unassigned in the WUZ system).

**Figure 6:**
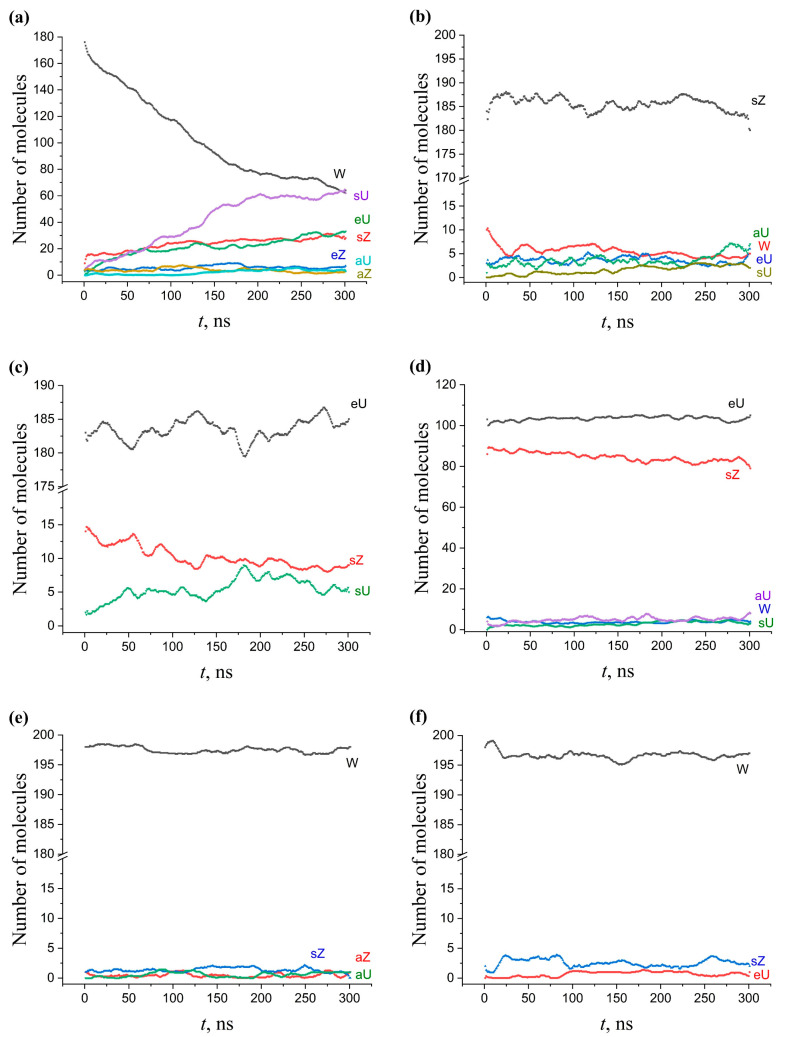
Conformation changes in the single-component membranes during 300 ns MD. (**a**) AMA-W, (**b**) AMA-sZ, (**c**) AMA-eU, (**d**) AMA-sZ+eU, (**e**) KMA-W, (**f**) MMA-W.

In contrast to the W conformation, other types of AMA conformations exhibit significantly greater stability. In particular, the conversion of the sZ and eU conformations occurs to a much lesser extent, or is not noticeable at all (Figure 6). The smallest changes occur in the membranes enriched in sZ structures.

#### 2.5.2. Single-Component KMA and MMA Membranes

In contrast to AMA, the W conformation of the single-component KMA and MMA membranes remains quite stable during 300 ns of evolution and very slowly transforms over time into other forms (or does not transform at all) (Figure 6). The reason for such a sharp difference in behavior may be that KMA and MMA have additional hydrophilic groups at the *c* and *d* points of their molecules. These groups are located at the membrane boundary in contact with water, which could stabilize the additional bending of the molecules. This leads to the fact that the thickness of the membranes is significantly smaller compared to the AMA membranes based on the sZ, eZ, eU, sU conformations. As will be shown below, this also affects the structure of the multicomponent membranes.

#### 2.5.3. Multicomponent Membranes

**Figure 7:**
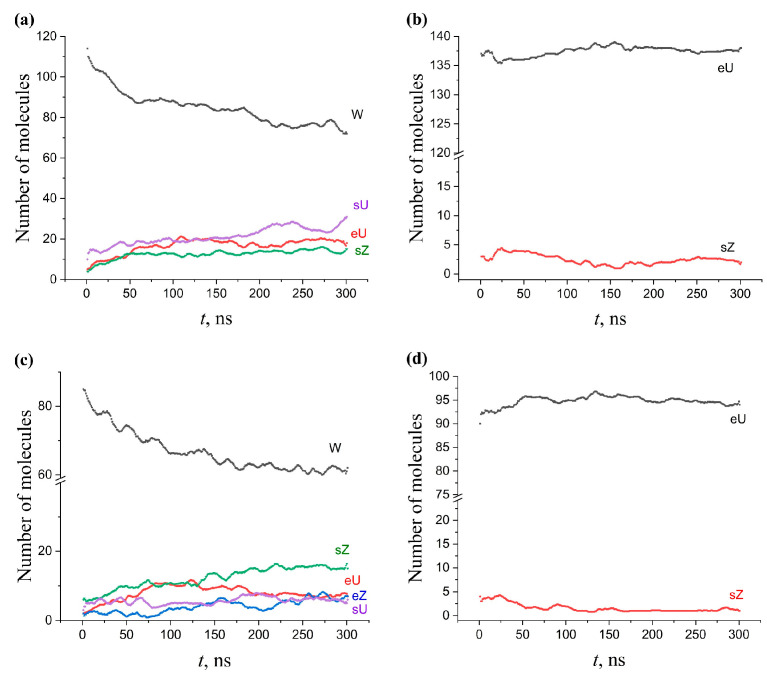
Conformational changes for AMA in the multicomponent AMA–KMA–MMA membranes during 300 ns MD. (**a**) Mixed W–W–W membrane with ratio of components 70:15:15. (**b**) Mixed eU–W–W membrane with ratio of components 70:15:15. (**c**) Mixed W–W–W membrane with ratio of components 50:25:25. (**d**) Mixed eU–W–W membrane with ratio of components 50:25:25.

The conformational changes that occurred during 300 ns of NPT evolution in multicomponent membranes initially built on the basis of the W conformation are shown in Figure 7. The comparison of Figure 7a,c (all components are of the W type) with Figure 6a (single-component AMA-W membrane) indicates that the transformation of the W conformation in a multicomponent membrane occurs significantly slower. An increase in the KMA+MMA content from 0 to 50% leads to a progressive stabilization of the W conformation. After 300 ns, only 32% of AMA molecules keep the W shape in the neat AMA membrane (64 out of 200 molecules, Figure 6a), compared to 51% in the 70:15:15 membrane (71 of 140, Figure 7a) and 62% in the 50:25:25 membrane (62 of 100, Figure 7c).

The same stabilizing effect of KMA/MMA, albeit less pronounced, appeared for the membrane built on the basis of AMA-eU. The comparison of Figure 7b,d with Figure 6b shows that AMA-eU in a mixed membrane has more stable behavior, with fewer by-product conformations, and the main conformation itself experiences much smaller fluctuations in its concentration. 

Thus, the presence of the KMA and MMA components, which tend to be in the W conformation, additionally stabilized the W conformation of AMA, but this effect was dependent on the KMA/MMA concentration. The dependence of the AMA-eU conformation stability on the addition of KMA/MMA probably was less pronounced because this conformation was also quite stable in a pure AMA-eU membrane. Increase of conformational stability of the AMA forms may be one of the functions of KMA/MMA in the mycobacterial cell wall. This could explain the fact that the bacterium builds a cell wall based on the composite AMA/KMA/MMA membranes. 

### 2.6. Effect of Structural Composition on the Thickness and Density of MA Membranes

**Figure 8:**
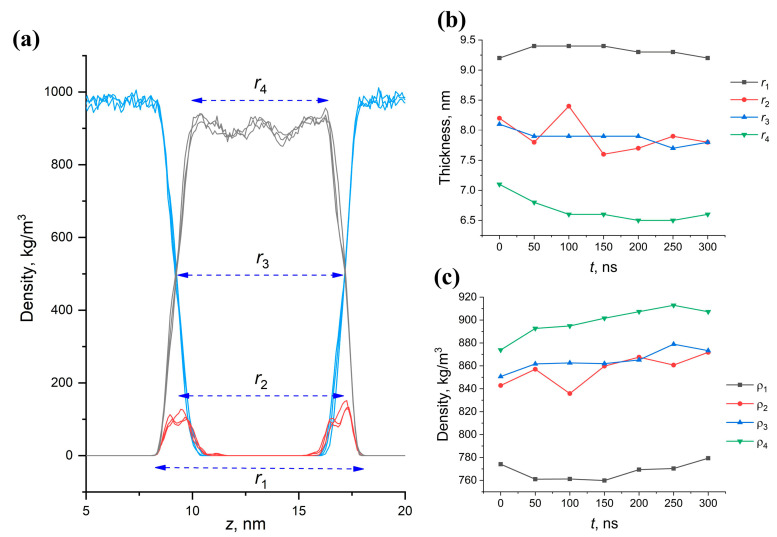
(**a**) Different definitions of membrane boundaries for the thickness and density evaluations. (**b**,**c**) Typical changes in the membrane thickness (**b**) and density (**c**) calculated with different definitions for the AMA-eU membrane.

It is interesting to consider the influence of the conformational composition on the density and thickness of the membrane. The thickness of the membrane, however, can be analyzed in different ways. In this paper, we have considered several ways to define this parameter. The general approach is to calculate the dependence of the membrane density on the transverse (*z*) coordinate. Figure 8a shows a typical density profile that is obtained in simulated membranes. In this plot, the values $r_{1}$–$r_{4}$ represent different ways of describing the thickness: $r_{1}$ is the distance between the extreme oxygen atoms of the hydrophilic groups, $r_{2}$ is the distance between the density maxima of oxygen atoms of the hydrophilic groups; $r_{3}$ is the distance at which the atomic densities of the solvent and membrane become equal; $r_{4}$ is the thickness of the dense part of the membrane, defined as the distance between the points at which the density of the membrane becomes equal to the average density calculated from the parameter *r*_3_. Four different ways of calculating thickness and density based on these distances lead to different absolute values, and these values behave differently in the course of temporal evolution. 

It can be seen that the evolution of the thickness values over time, determined from $r_{1}$ and $r_{4}$, differed greatly from each other, while the parameters $r_{2}$ and $r_{3}$ produced similar values (Figure 8b,c). At the same time, the thickness based on $r_{3}$ was more stable during time evolution, while $r_{2}$ experienced significant random fluctuations. In addition, $r_{3}$ was always somewhere in the middle between the values of $r_{1}$ and $r_{2}$. Thus, the most reliable measures of the membrane thickness are the parameters based on $r_{3}$, while $r_{1}$ and $r_{4}$ characterize the upper and lower limits of the determined values.

Thus, the thickness and density of the membranes of various component and conformational compositions after 300 ns MD were evaluated from $r_{3}$ value. The most noticeable feature of the data (Table 2) is that the membrane in which AMA prevails in the eU conformation is much thicker (which is not surprising, given the elongation of the molecules in this conformation) and, at the same time, much denser. It can be said with certainty that such a packing of MA molecules in the membrane promotes the formation of a much stronger membrane of the outer shell of the mycobacterium, which will be much more resistant to external factors.

As noted above, an increase in the concentration of the KMA and MMA components in the multicomponent membranes leads to a strong decrease in the thickness and density of the membrane. As can be seen from Table 2, the transition from a pure AMA-eU membrane to a similar membrane with 30% (140eU–30W–30W) or 50% (100eU–50W–50W) content of other components causes a decrease in thickness from 7.8 to 5.9 and 5.1 nm, which cannot but affect the strength of the bacterial membrane. Thus, apparently, KMA and MMA in bacterial membranes play the role of the AMA conformational stabilizer, increasing the stability of membranes over time. It could also create a molecular mechanism allowing the mycobacterium to modulate the envelope properties over its lifetime by synthesizing the KMA and MMA components in greater or lesser amounts.

**Figure 9:**
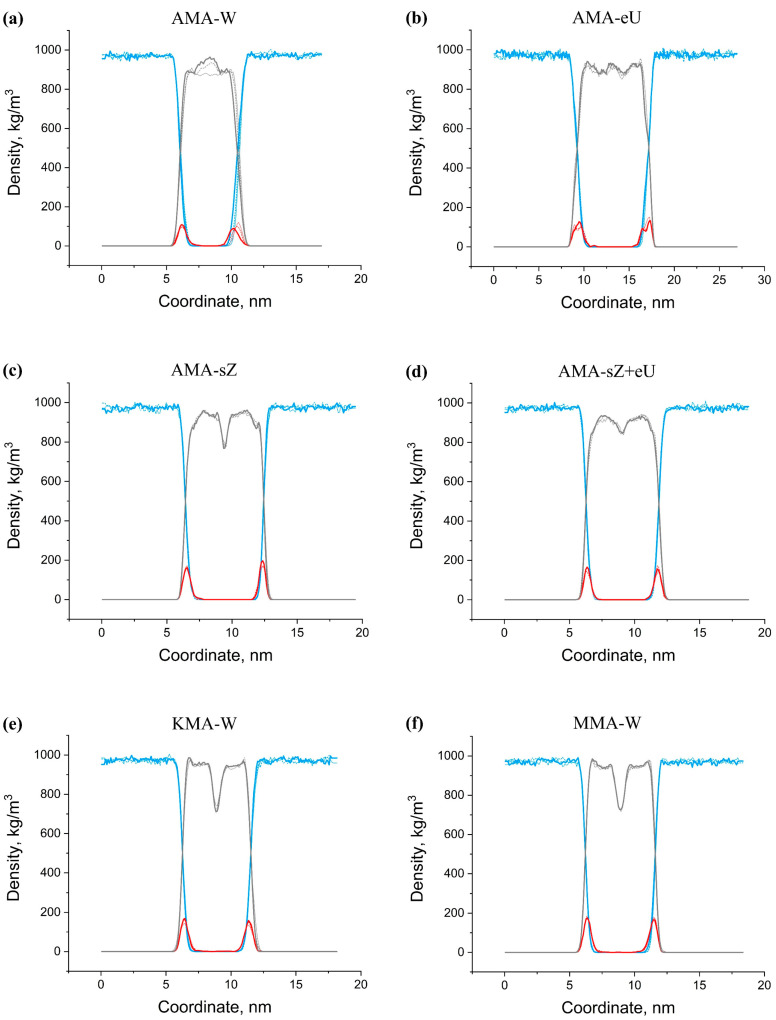
Density profiles in the single-component membranes: water (blue line); oxygen atoms (red line); all atoms of MA (gray line) at 100 ns (dotted line) 200 ns (dashed line); 300 ns (solid line). Membranes: (**a**) AMA-W, (**b**) AMA-eU, (**c**) AMA-sZ, (**d**) AMA-sZ+eU, (**e**) KMA-W, (**f**) MMA-W.

**Figure 10:**
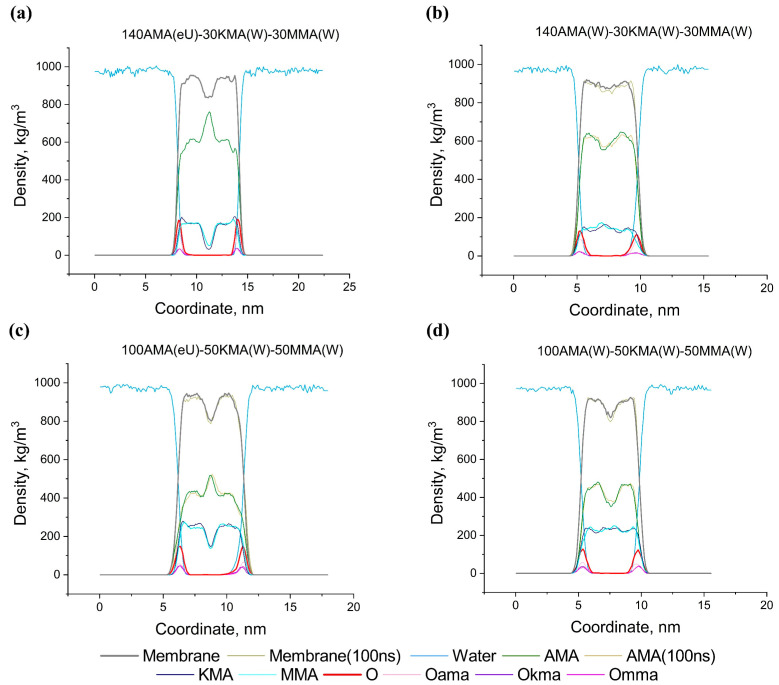
Density profiles for the multicomponent membranes at 100 and 300 ns.

Figure 9 shows the density profiles of the solvent atoms, MA atoms, and oxygen atoms of hydrophilic groups in the single-component membranes, and Figure 10 – in the multicomponent membranes. An analysis of these distributions allows us to note several features of different types of membranes. All types of membranes, including AMA-W, maintain their thickness and density for a long time. In the course of the 300 ns time evolution, no significant changes in the thickness of the membranes could be noticed. As can be seen from Figure 9a, even long-term time evolution during 1200 ns does not lead to a noticeable change in thickness. Changes in the other membranes are even less noticeable (Figure 9b–f) after 300 ns. This might indicate that this process occurs on a much longer time scale. Thus, apparently, the method of membrane synthesis affects the strength and thickness of a mycobacterial cell wall. It is possible that the synthesis method might change during the life of a bacterium depending on the environmental conditions. It is also possible that over time, under unfavorable conditions, the “maturation” of the cell wall could occur, involving slow formation of a stronger and thicker shell of the eU type. The AMA-W type membranes are characterized by compaction of the inner part of the membrane over time (density peak at the center of the membrane in Figure 9a). In the course of temporal evolution, this peak is rising, while the membrane thickness remains almost unchanged. Single-component membranes enriched in AMA-sZ conformations, on the contrary, have a density minimum in the central part (Figure 9b–d). Such membranes would obviously tend to delaminate and create solvent lenses in their core. A similar feature is observed in the single-component membranes based on KMA-W and MMA-W (Figure 9e,f), while this is not the case for AMA-W membranes.

Figure 10 shows the density profiles in multicomponent membranes of the eU–W–W and W–W–W initial compositions (the letters denote the initial conformations of AMA, KMA, MMA, respectively) at different contents of the KMA/MMA components (70% and 50% AMA). Figures S2–S7 in the Supplementary Materials show the spatial positions and shapes of separate KMA and MMA molecules in the multicomponent membranes. A distinctive feature of these systems is that in the case of eU–W–W membranes, the AMA component has a density maximum in the central part (Figure 10a,c), similar to the AMA-W and AMA-eU single-component membranes. At the same time, the KMA and MMA components are concentrated in the outer region of the membranes. Apparently, this is due to the fact that, for the KMA and MMA molecules, the W conformation is more stable because of the presence of additional hydrophilic groups in positions *d*, and it is favorable for these molecules to be located in the near-surface layer of membranes. However, for membranes of the W–W–W type (Figure 10b,d), the density profile is reversed: AMA in the central part has a minimum, and KMA/MMA has a maximum. At the same time, the total density profile of W–W–W has a much less pronounced minimum in the central part than in the case of eU–W–W. This is apparently explained by the fact that the elongated structure of AMA-eU does not allow shorter KMA/MMA molecules to approach each other, which leads to a lack of density in the center. In the environment of shorter AMA-W molecules, the KMA/MMA molecules approach each other much closer and create an increased density in the center. Interestingly, in this case, AMA has a reduced density in the central part, possibly due to its more compact near-surface packing or more oblate molecular ellipsoids. In the case of mixed W–W–W membranes, the different content of KMA/MMA almost does not change the physical characteristics (thickness, density, surface density) of the membranes compared to a pure AMA-W membrane. At the same time, increasing the content of KMA/MMA in the eU–W–W mixed membranes reduces the thickness, density and surface density. Nevertheless, all eU–W–W membranes are still stronger and thicker than the W–W–W and AMA-W membranes.

## 3. Materials and Methods

### 3.1. Initial Membrane Structures

Several types of bilayer membranes comprising 200 MA molecules (100 molecules in each leaflet) were selected for modeling: the single-component membranes of AMA, KMA, MMA molecules and two types of the three-component membranes AMA:KMA:MMA with different compositions (the ratio of molecules was 70:15:15 and 50:25:25 in each leaflet). Each system was built from the molecules of a certain conformation in order to assess the degree of stability or the rate of transformation of this conformation into others. Molecular orientations were chosen such that the hydrophilic group was in contact with the aqueous phase. However, not all conformations are able to satisfy this requirement (Figure 1), so the bilayers were built only on the basis of the conformations W, sZ, and eU. The membrane was formed using a special program that ensured packing of molecules by the simulated annealing method to a density of about 400–450 kg/m^3^ while a real density of mycolic acids is 800–900 kg/m^3^. The membranes were subjected to additional densification by repeating the cycles of NVT and NPT evolution, compression, and relaxation (see the MD simulation protocol below). Although the membranes initially represented a system of identical conformations, some of the conformations changed during compaction, and the production run was already performed with a system that was a mixture of the initial conformation (about 90% of the molecules) and a number of some other conformations (usually 1–5% each). 

### 3.2. MD Simulation Protocols

For the molecular dynamics simulations, the all-atom CHARMM36 force field [30] was used, which belongs to the modern family of force fields, continues to be actively improved, and is recommended for modeling lipid membranes [31]. The topologies for the MA molecules were obtained using the CHARMM-GUI automatic topology builder [32,33]. A rectangular box with three-dimensional periodic boundary conditions was used. The box size was chosen so that the amount of added water was about 35 thousand molecules. The number of atoms in the simulated membrane plus water (water model SPC216) systems was 140–160 thousand. The simulation was carried out in the GROMACS 2022.2 software package [34] with the NVIDIA GeForce RTX 3080Ti GPU in several stages: (1) energy minimization (up to *F_max_* ≤ 1000 kJ/mol); (2) NVT ensemble with the “frozen” backbone heavy atoms of acids (*t* = 100 ps, v-rescale thermostat, *T* = 300 K); (3) NPT ensemble with the “frozen” acid nuclei (*t* = 100 ps, v-rescale thermostat, Parrinello-Rahman barostat); (4) 20 cycles of compression and relaxation of the membrane (150 and 350 ps at a lateral pressure of 1.2 and 1 bar, respectively). The algorithm presented above ensures the accurate gradual compression of the membrane so that no cavities or pockets of solvent remain inside the bilayer, and the MA system does not collapse into a “blob”. As a result, the MA bilayers were obtained with the thickness of 3.5–7.5 nm and the density about 800–900 kg/m^3^. These systems were used for the production runs in the NPT ensemble for at least 300 ns at a pressure of 1 bar, T = 300 K. For the AMA system with the W conformation, the simulation was performed for 1200 ns in order to achieve an equilibrium concentration of structures after conversion of the parent conformation. 

### 3.3. Conformations of Mycolic Acids

To describe the conformations of mycolic acids, following the approach [18], we considered the distances between points *a*, *b*, *c*, *d*, *e* located at the ends of the aliphatic chain and on the atoms of the functional groups (Figure 1). Within the WUZ classification, different arrangements of the four fragments on the plane between these points (i.e., different ways of chain bending) create 7 types of conformations W, aZ, sZ, eZ, aU, sU, eU, while the remaining molecules belong to the structure I (Straight). Although the bending of the MA molecules in vacuum requires practically no energy and does not involve overcoming any noticeable energy barriers, inside a close-packed layer these conformations are structurally and thermodynamically not equivalent, and their predominant content in the membrane leads to differences in its thickness, density, and other properties. The set of 7 structures, obviously, does not exhaust all possible conformations since, firstly, chain fragments can go out of plane when bent and, secondly, they could bend not only at the points *a–e*. The work [20] also considers other conformations, including knotted structures of complex topology. 

To reveal the MA conformations formed in the system, an MD trajectory (typically 300 ns long) was analyzed, with frames recorded every 1 ns. The structures of all MA molecules in each frame were processed using the open-source, community-developed PLUMED library version 2.7.2 [35,36], which calculates the distances between the points *a*, *b*, *c*, *d*, *e* selected to match certain reference atoms in the MA molecules. The conformation boundary values for the defining distances *ac*, *ae*, *ce*, *bd* were slightly changed compared to [18] in order to ensure better agreement with the visual assignment of conformations. The standard approach [18] does not allow all molecular structures to be assigned to seven types of conformations, and approximately 30–35% of the unclassified (Straight) structures remain. However, many of these structures turn out to be structurally very similar to one of the WUZ conformations, and the quality of assignment can be improved by additional classification of these structures using the *k*-means clustering algorithm with preliminary assignment of cluster centers. A detailed description of the algorithm for selecting cluster centers, as well as a table of boundary distances used, are given in the Supplementary Materials (Section S1 “Algorithm for the refinement of conformation identification” and Table S1).

The post-MD analysis of the obtained MD trajectories was performed using the standard GROMACS utilities as well as a set of in-house software tools for conformational analysis, density calculations, and kinetic parameters evaluations. These programs can be found on GitHub [37] or obtained by request from the authors. 

## 4. Conclusions

Our results demonstrate that the structure and properties of the bilayer mycolic acid membranes strongly depend on the way the AMA molecules were initially packed, as well as on the presence of the KMA and MMA components. Membranes with different initial structures can retain their thickness, density distribution and, in some cases, conformational composition for a long time (durations about 300 ns or, in the case of the AMA-W membrane, up to 1.2 μs, were achieved in the MD simulations).

For the AMA-based membranes, the most labile conformation was W, which changes significantly within 300 ns, turning into other types of conformations. In contrast to the W structure, the other types of initial packing studied (sZ and eU, as well as their mixture) turn out to be much more stable, and the product conformations that accumulate over 300 ns do not exceed 10%.

The conformational transitions that occurred in the AMA membranes based on the W conformation were described by the first-order kinetics, with the decay of the W structure leading mainly to the sU, eU, sZ structures with noticeably higher amount of sU. The characteristic time of the W decay and the accumulation of product conformations was 160–220 ns.

In contrast to AMA, the W conformations in the KMA and MMA single-component membranes remained stable during 300 ns of evolution and were very slowly transformed over time into other forms, apparently due to the fact that these molecules have additional hydrophilic groups at point *d*.

In the multicomponent membranes, the presence of the KMA and MMA components, which tend to be in the W conformation, additionally stabilized both the W and eU conformations of AMA, and this effect depended on the KMA/MMA concentration. It is possible that KMA and MMA in the mycobacterial envelope play the role of a conformational stabilizer of AMA, increasing the stability of membranes over time. It could also create a molecular mechanism allowing the mycobacterium to modulate the envelope properties over its lifetime by synthesizing the KMA and MMA components in greater or lesser amounts.

The membrane where AMA mostly has the eU conformation is much thicker and, at the same time, much denser. It can be said with certainty that such a packing of MA molecules in the membrane promotes the formation of a much stronger outer mycobacterial membrane that should be much more resistant to the threatening external factors.

## Figures and Tables

**Table 1:** Kinetic characteristics of the conformational transformations in the AMA-W system during 1200 ns of NPT MD.

	Most Abundant Conformations *				
	W	sZ	eU	sU	aU
Final number of molecules $N_{1200}$	33	36	38	87	3
Equilibrium number of molecules $N_{\infty }$	35.3 ± 0.1	34.2 ± 0.1	35.6 ± 0.1	85.6 ± 0.1	5.25 ± 0.04
Equilibrium fraction of molecules $x=N_{\infty }/N_{total}$	0.177	0.171	0.178	0.428	0.027
Characteristic time of accumulation/decay τ, ns	192 ± 0.1	198.5 ± 3.6	222.6 ± 5.8	220.1 ± 2.1	160.7 ± 6.6
Pre-exponential coefficient A	135.0 ± 0.4	−20.2 ± 0.2	−26.8 ± 0.3	−83.6 ± 0.4	−6.6 ± 0.2
Rate constant of accumulation/decay $\left(1/\tau \right)\cdot 10^{–6}$, s^–1^	5.20	5.04	4.49	4.54	6.22
Coefficient of determination $R^{2}$	0.991	0.924	0.863	0.979	0.684

* Exponential model failed to describe the evolution of the eZ and aZ conformations due to the small number of molecules in a sample (2 and 1 molecule, respectively).

**Table 2:** Thickness and density of the AMA-based and mixed membranes after 300 ns of NPT MD (parameters determined on the basis of $r_{3}$ ).

Membrane	Thickness, nm	Density, kg/m^3^	Surface Density, Molecules/nm^2^
AMA-W	4.4	857.5	2.16
AMA-W (1200 ns)	4.4	862.7	2.16
AMA-eU	7.8	907.3	3.84
AMA-sZ	5.9	891.5	2.93
AMA-(sZ+eU)	5.6	865.6	2.69
KMA-W	5.1	901.3	2.31
MMA-W	5.3	893.9	2.40
140eU–30W–30W ^1^	5.9	898.2	2.86
140W–30W–30W ^1^	4.6	861.8	2.17
100eU–50W–50W ^1^	5.0	876.8	2.38
100W–50W–50W ^1^	4.5	865.6	2.12

^1^ *n*X–*m*W–*m*W: bilayer membranes constructed from *n* AMA-X (X = W or eU), *m* KMA-W, and *m* MMA-W molecules (equal numbers of all conformations in each leaflet).

## Data Availability

The data presented in this study are available in the article or supplementary materials.

## Supplementary Materials

The following supporting information can be downloaded at: https://www.mdpi.com/article/10.3390/molecules28031347/s1, Section S1: Algorithm for the refinement of conformation identification; Table S1: Bounds of intramolecular distances for determining WUZ conformations; Figure S1: Changes in the average massless gyration radii and average hydrodynamic radii of AMA, KMA, MMA in the membranes of different initial structure during MD simulation; Figures S2–S7: Mutual arrangement of MMA and KMA in the mixed membranes 140AMA(W):30MMA:30KMA and 140AMA(eU):30MMA:30KMA for the initial configuration (0), 100, 200 and 300 ns of molecular dynamics simulation.
