# Supplementary material for: Conformational Dynamics and Stability of Bilayers Formed by Mycolic Acids from the Mycobacterium tuberculosis Outer Membrane
Source: Molecules. 2023 Jan 31;28(3):1347. doi: 10.3390/molecules28031347 (PMC9921641; doi:10.3390/molecules28031347)
Supplement: Supplementary file 1 [file molecules-28-01347-s001.zip › molecules-2109574-supplementary.pdf]

# Supplementary Materials

# Conformational dynamics and stability of bilayers formed by mycolic acids from the *Mycobacterium tuberculosis* outer membrane

**Liana A. Savintseva [1], Ilya S. Steshin [1], Alexander A. Avdoshin [1], Sergey V. Panteleev [1], Alexey V. Rozhkov [1], Ekaterina A. Shirokova [1], Grigory D. Livshits [1], Alexander V. Vasyankin [1], Eugene V. Radchenko [1,2], Stanislav K. Ignatov [1,*] and Vladimir A. Palyulin [1,2,*]**

[1] Department of Chemistry, Lobachevsky State University of Nizhny Novgorod, Nizhny Novgorod 603022, Russia
[2] Department of Chemistry, Lomonosov Moscow State University, Moscow 119991, Russia

TABLE OF CONTENT


| No. | Item | Page |
|---|---|---|
| 1 | Section S1: Algorithm for the refinement of conformation identification. | 2 |
| 2 | Table S1: Bounds of intramolecular distances for determining WUZ conformations. | 2 |
| 3 | Figure S1: Changes in the average massless gyration radii (*a*) and average hydrodynamic radii (*b*) of AMA, KMA, MMA in the membranes of different initial structure during MD simulation. | 4 |
| 4 | Figures S2–S7: Mutual arrangement of MMA and KMA in a mixed membranes 140AMA(W):30MMA:30KMA and 140AMA(eU):30MMA:30KMA for the initial configuration (0), 100, 200 and 300 ns of molecular dynamics simulation. | 5–14 |

**Section S1. Algorithm for the refinement of conformation identification**

The assignment of the structures obtained during MD modeling to one of the seven WUZ conformations was carried out based on the comparison of the distances *ac, ae, ce* and *bd* with the boundary distances given in Table S1.

**Table S1.** Bounds of intramolecular distances for determining WUZ conformations.

| Conformation | Boundary distances, nm | | | |
|---|---|---|---|---|
| | *ac* | *ae* | *ce* | *bd* |
| W | < 2.3 | < 2.8 | < 2.0 | < 1.3 |
| aZ | > 2.3 | > 2.8 | < 2.0 | < 1.3 |
| eZ | < 2.3 | > 2.8 | > 2.0 | < 1.3 |
| sZ | < 2.3 | < 2.8 | < 2.0 | > 1.8 |
| eU | < 2.3 | > 1.5 | > 2.0 | > 1.8 |
| sU | > 2.3 | < 2.8 | > 2.5 | < 1.6 |
| aU | > 3.0 | > 2.8 | < 1.8 | > 1.8 |

The use of the boundary distances given in Table S1 makes it possible to definitely classify about 65% of the structures formed during MD evolution. The remaining structures formally belong to the Straight conformation, but in reality, often resemble one of the seven WUZ conformations. Therefore, for their additional analysis and assignment, the *k*-means clustering algorithm was used with a preliminary determination of the cluster centers.

Cluster centers for conformations $\alpha$ ($\alpha$ = W, sZ, eU) were calculated as the average values of the distances $<ac>_{\alpha}$, $<ae>_{\alpha}$, $<ce>_{\alpha}$, $<ed>_{\alpha}$ over all molecules of conformation α that were already classified using boundary distances for sampling structures from the MD trajectory at the simulation time of 100 ns. The choice of time is due to the need to accumulate a sufficient population of structures in different conformations and achieve their structural diversity relative to the initial state.

The number of structures assigned to the types aZ, eZ, sU, aU was usually not enough to find the hypothetical cluster centers in a similar way with the required accuracy. The method of additional extrapolation was used to find the centers of these clusters. To do this, from the coordinates of the centers of clusters W, sZ and eU and the boundaries of intramolecular distances *ac, ae, ce, ed* (Table S1) of conformations W, sZ and eU, three 4-dimensional vectors $v_{\alpha}$ were formed by the formula:

$$v_{\alpha} = (|<ac>_{\alpha} - ac_{\alpha}|, |<ae>_{\alpha} - ae_{\alpha}|, |<ce>_{\alpha} - ce_{\alpha}|, |<bd>_{\alpha} - bd_{\alpha}|),$$

which were then averaged over three values of $\alpha$ ($\alpha$ = W, sZ, eU). The components of the averaged vector $<v>$ correspond to the approximate margins from the cluster center to the distance boundaries of the corresponding conformations. These margins can be used to find unknown cluster centers.

For this purpose, the vector <*v*> was combined with the coordinates of the boundaries of the corresponding conformation $\beta$ ($\beta$ = aZ, eZ, sU, aU): if the value of the boundary distance in Table S1 is preceded by the sign <, then the corresponding coordinate of the vector <*v*> was subtracted from this value; if there is a > sign, then it was added to it. The resulting vector $c_\beta$ determines the approximate coordinates of the $\beta$ cluster center. These coordinates are then passed to the *k*-means clustering algorithm.

$$c_\beta = (ac_\beta \pm ac_{<v>}, ae_\beta \pm ae_{<v>}, ce_\beta \pm ce_{<v>}, bd_\beta \pm bd_{<v>})$$

This procedure can be illustrated by the following scheme (for simplicity, only two distances $d_1$, $d_2$ out of four defining distances *ac, ae, ce, bd* are shown). Solid black lines represent the distance boundaries. Blue and red crosses show the cluster centers for the two out of three abundant conformations (e.g., W, sZ, or eU). For them, the $v_\alpha$ vectors are ($r_1$, $r_2$) and ($r_3$, $r_4$). The averaged vector components <*v*> = ($r_5$, $r_6$) define the offsets of the unknown center $c_\beta$ (green triangle) for the $\beta$ cluster from the respective distance boundaries ($\beta$ is one of aZ, eZ, sU, aU).

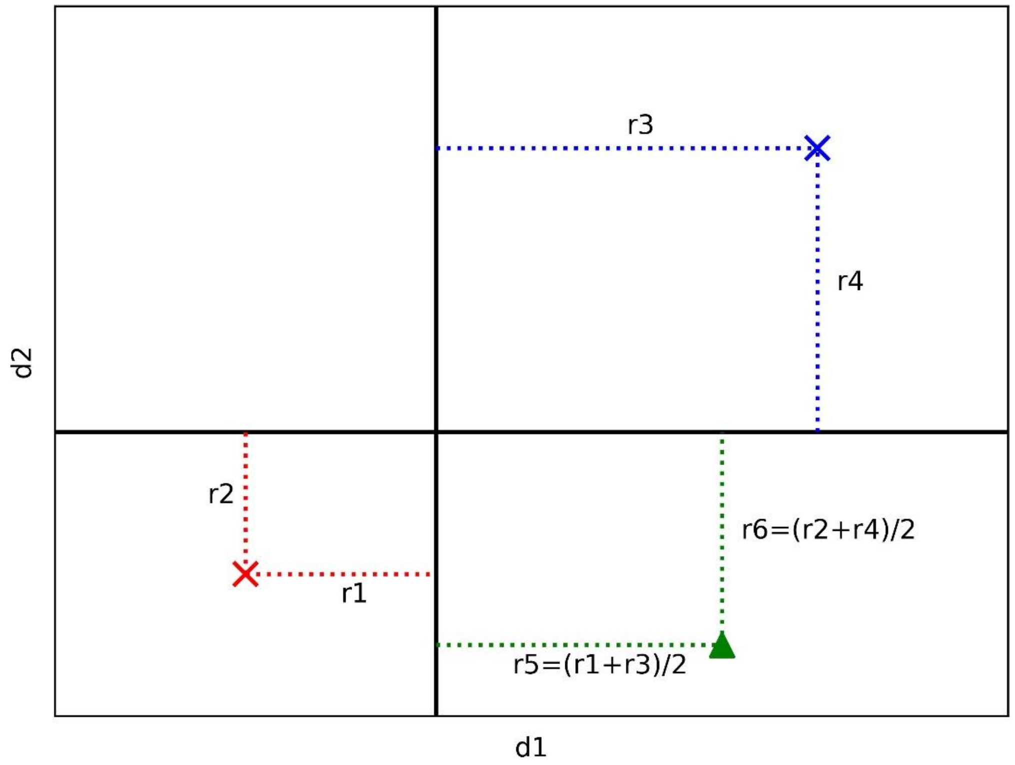

(*a*)

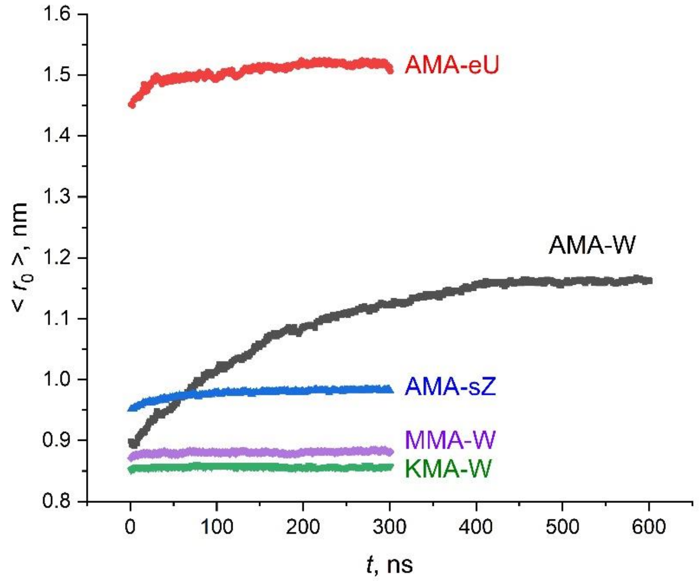


(*b*)

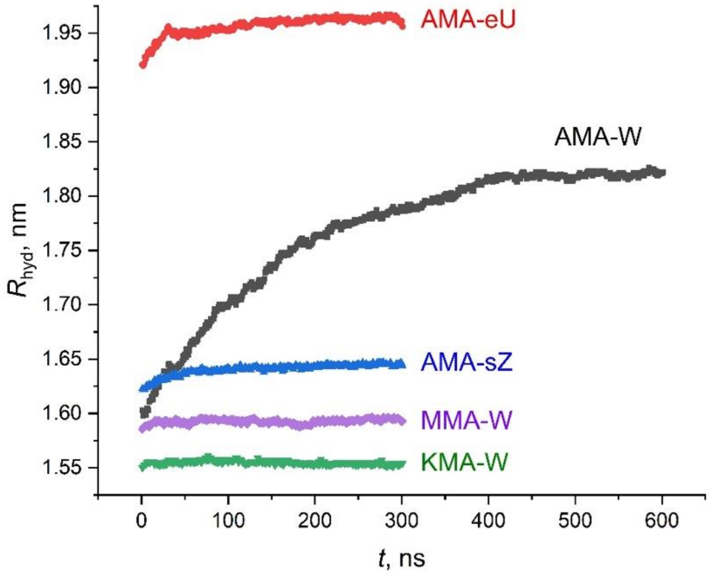


**Figure S1.** Changes in the average massless gyration radii (*a*) and average hydrodynamic radii (*b*) of AMA, KMA, MMA in the membranes of different initial structure during MD simulation.

Side view

Top view

0 ns

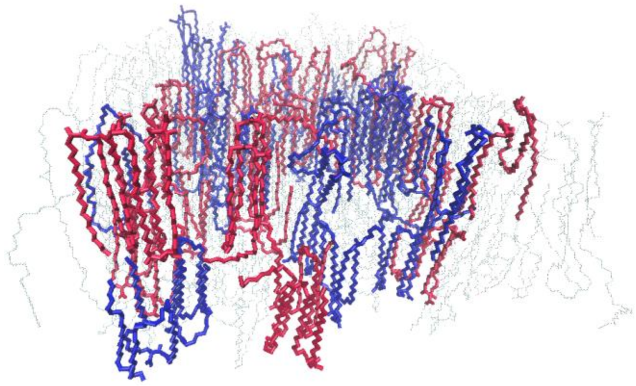

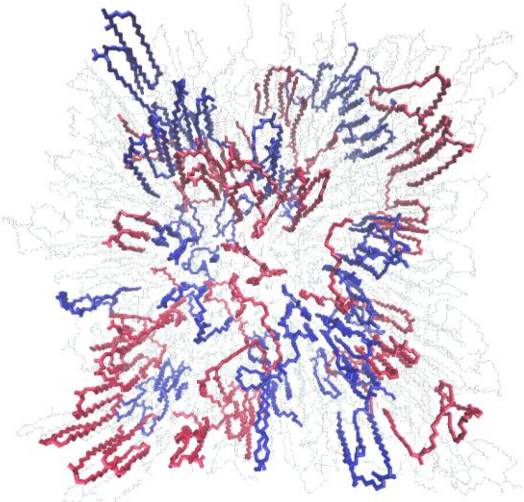

100 ns

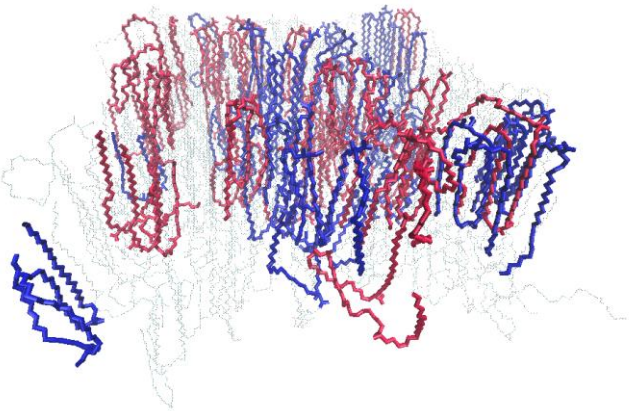

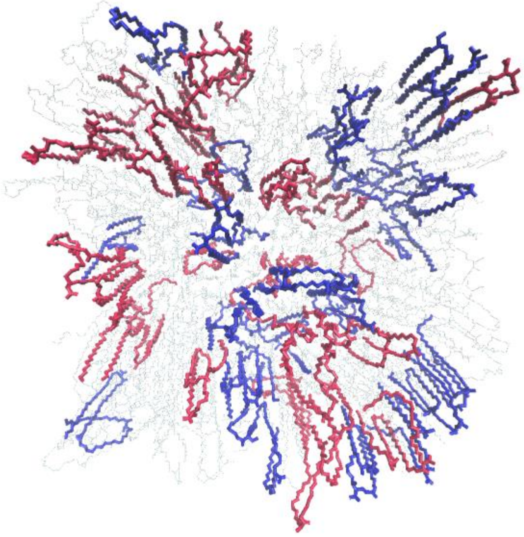

200 ns

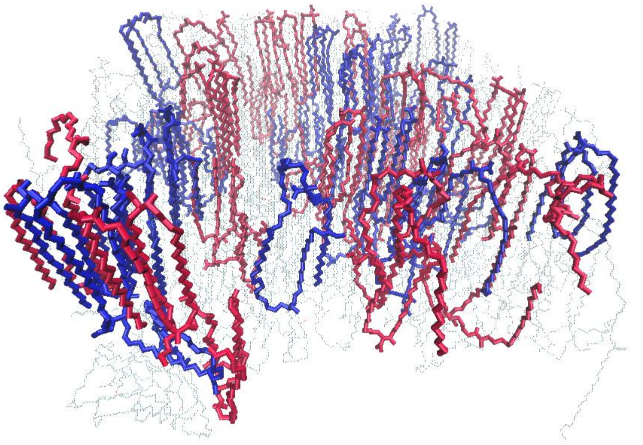

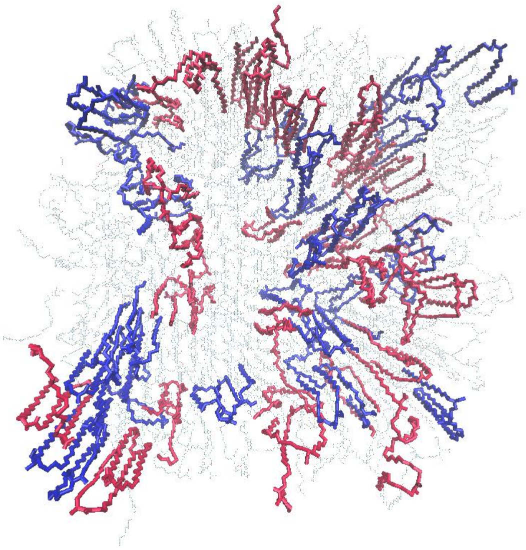

300 ns

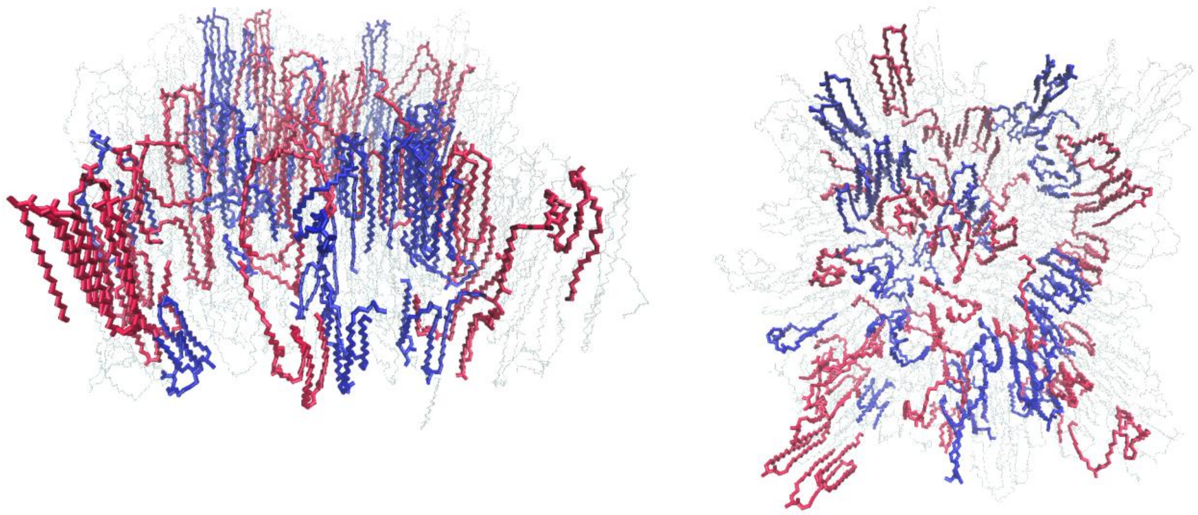

**Figure S2.** Mutual arrangement of MMA and KMA in a mixed membrane 140AMA(W):30MMA:30KMA for the initial configuration (0), 100, 200 and 300 ns of molecular dynamics simulation. MMA molecules are highlighted in blue, KMA – in red. Weak halftone background represents the AMA molecules with W conformation.

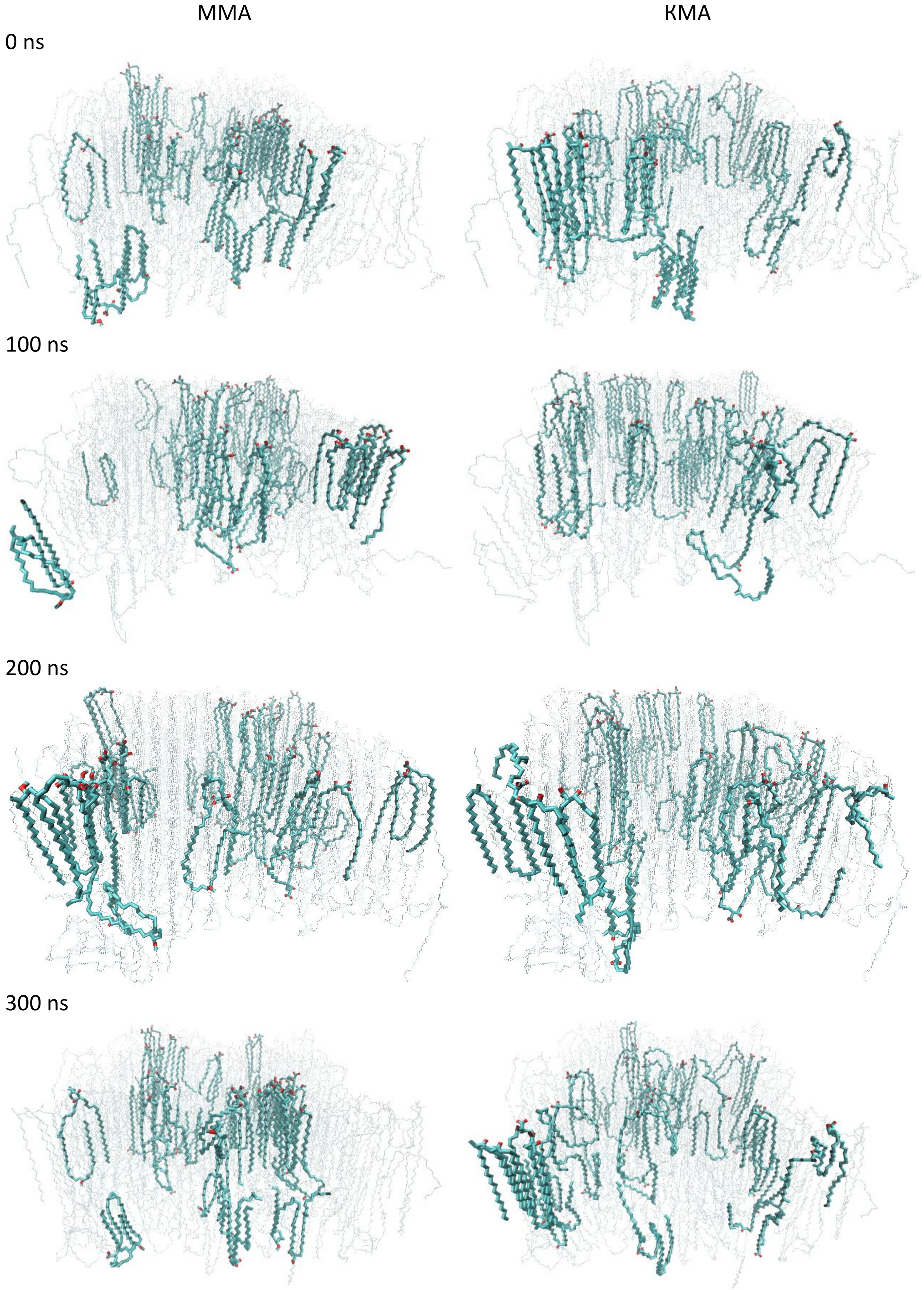


**Figure S3.** Conformations of MMA and KMA in a mixed membrane 140AMA(W):30MMA:30KMA for the initial configuration (0), 100, 200 and 300 ns of molecular dynamics simulation. Weak halftone background represents the AMA molecules with W conformation. Side view.

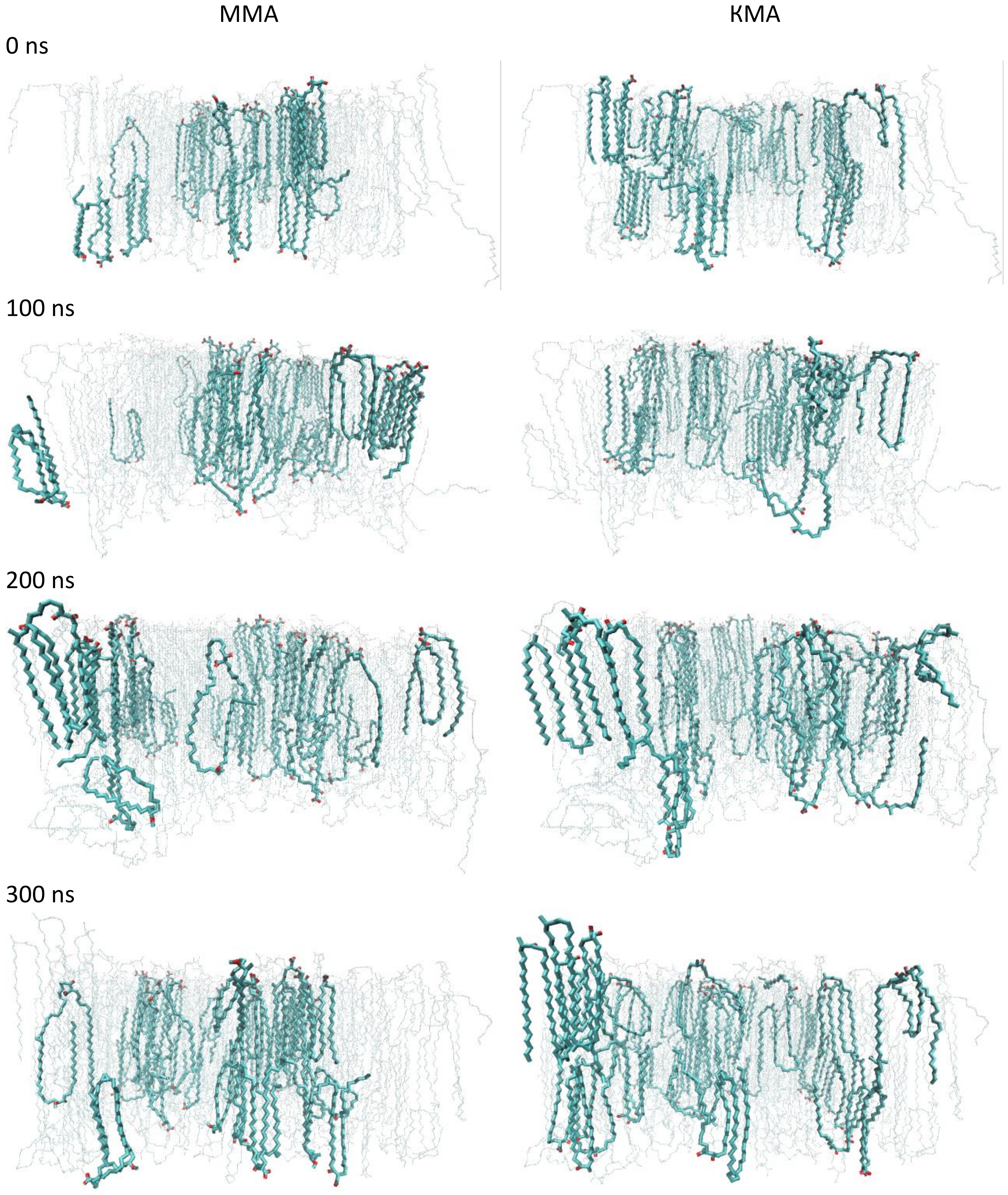


**Figure S4.** Conformations of MMA and KMA in a mixed membrane 140AMA(W):30MMA:30KMA for the initial configuration (0), 100, 200 and 300 ns of molecular dynamics simulation. Weak halftone background represents the AMA molecules with W conformation. The observation point passes through the XOY plane.

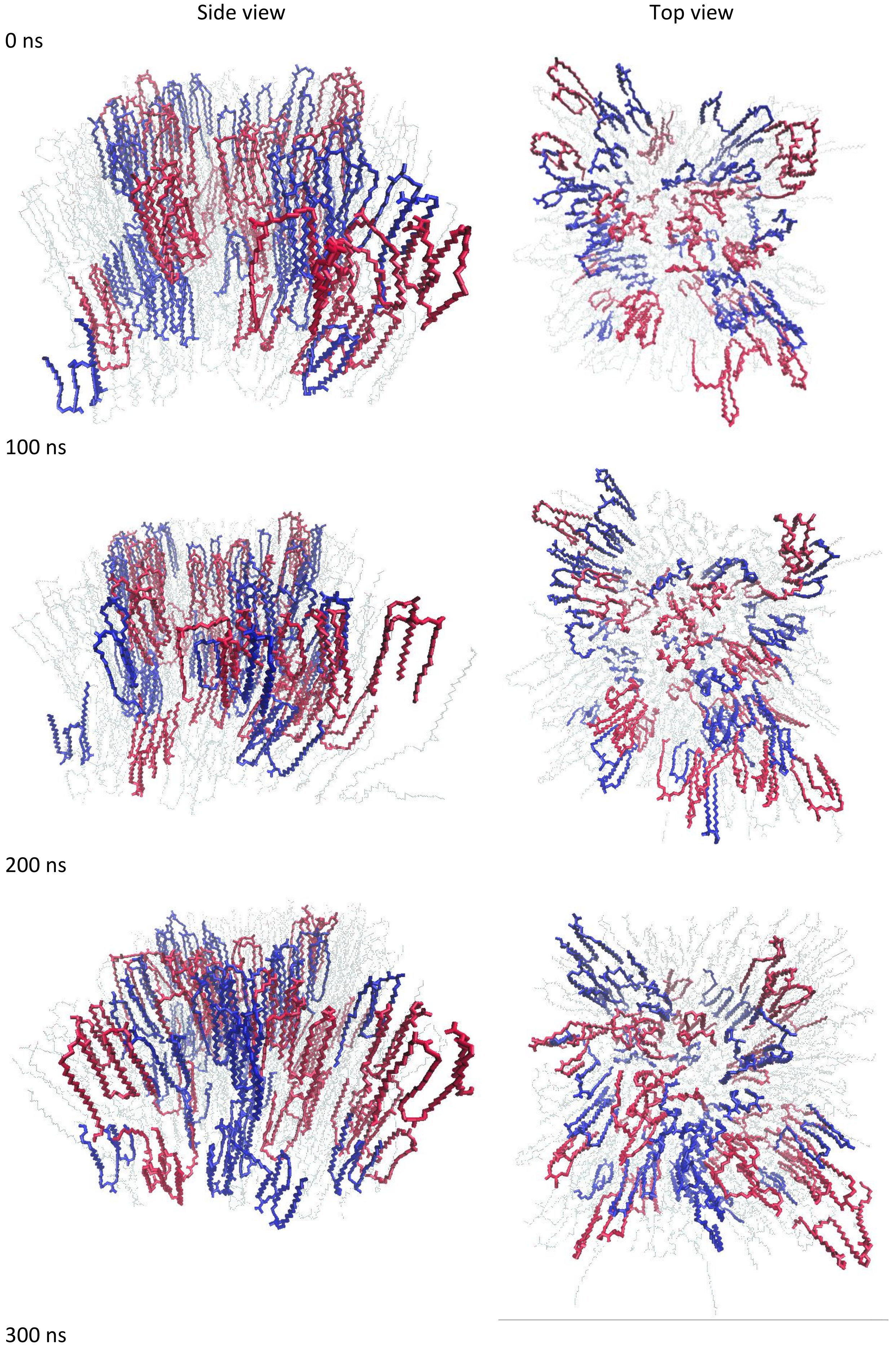
Side view
Top view
0 ns
100 ns
200 ns
300 ns

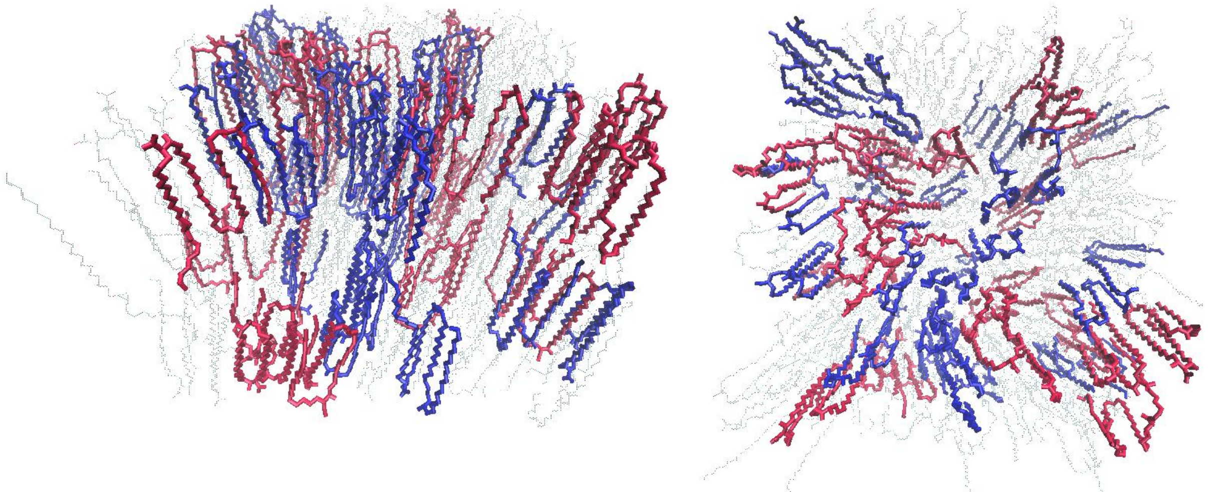

**Figure S5.** Mutual arrangement of MMA and KMA in a mixed membrane 140AMA(eU):30MMA:30KMA for the initial configuration (0), 100, 200 and 300 ns of molecular dynamics simulation. MMA molecules are highlighted in blue, KMA – in red. Weak halftone background represents the AMA molecules with eU conformation.

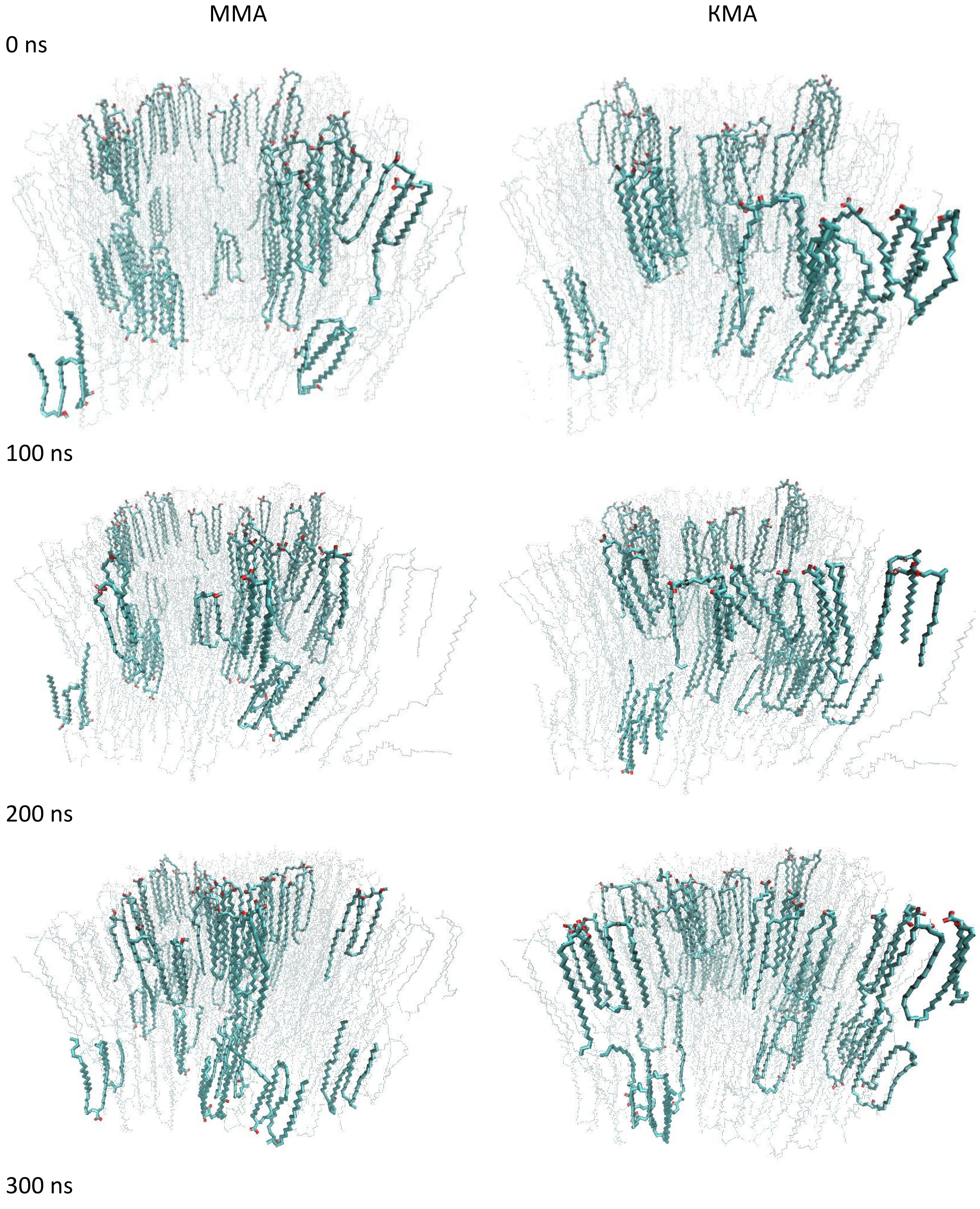
MMA
KMA
0 ns
100 ns
200 ns
300 ns

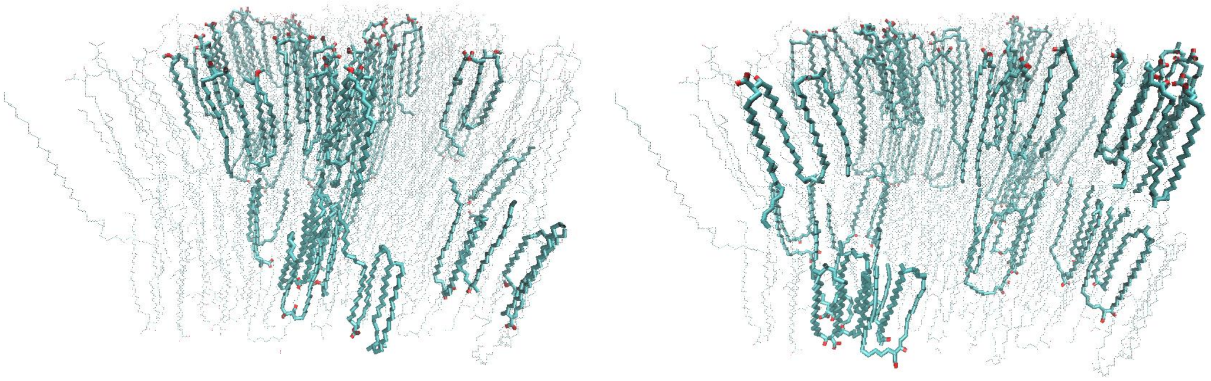

**Figure S6.** Conformations of MMA and KMA in a mixed membrane 140AMA(eU):30MMA:30KMA for the initial configuration (0), 100, 200 and 300 ns of molecular dynamics simulation. Weak halftone background represents the AMA molecules with eU conformation. Side view.

MMA KMA

0 ns

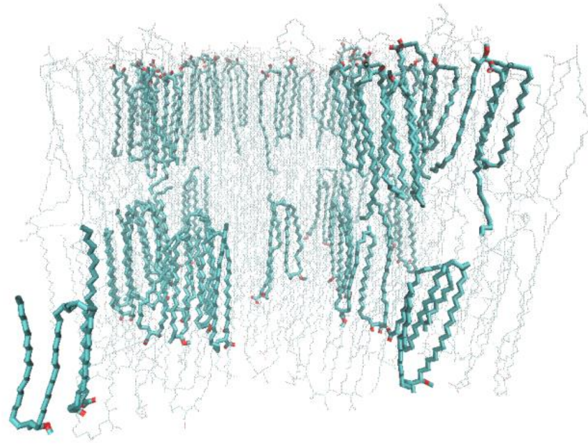

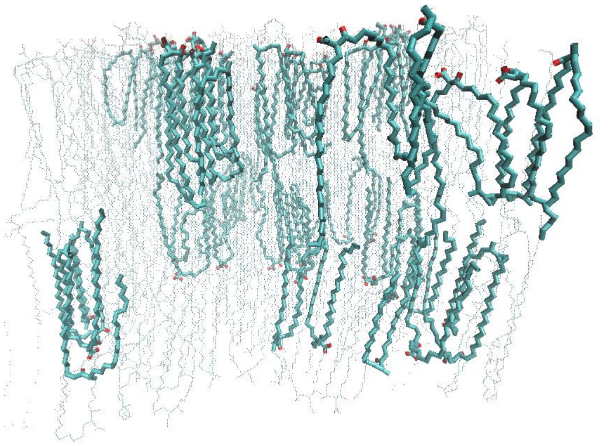

100 ns

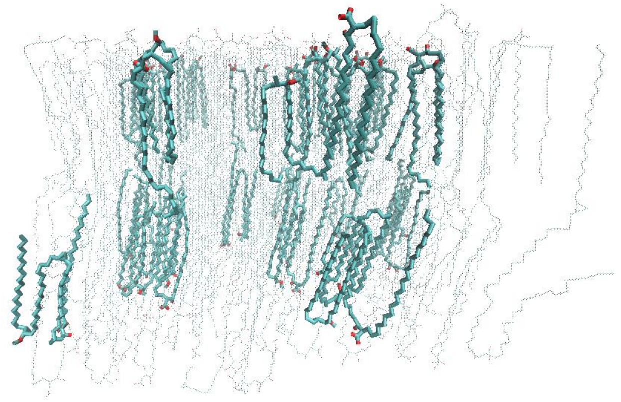

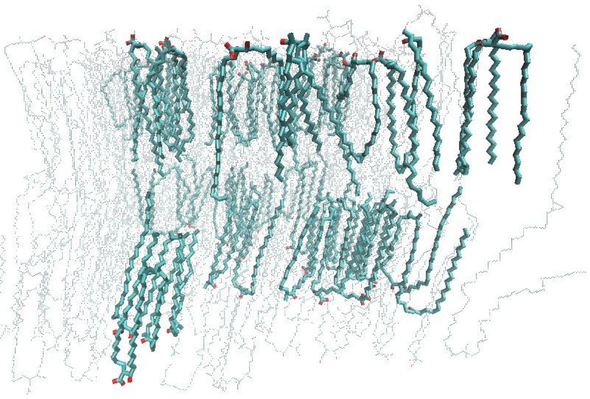

200 ns

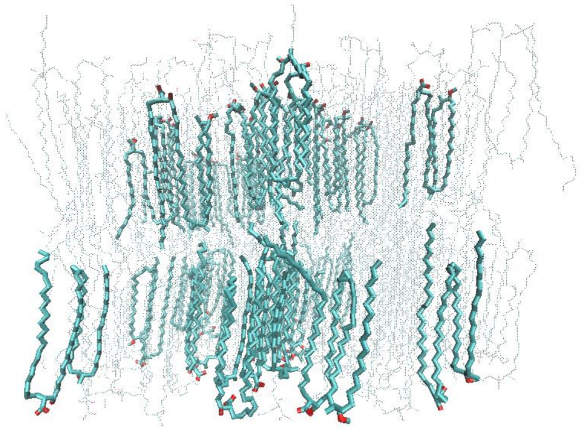

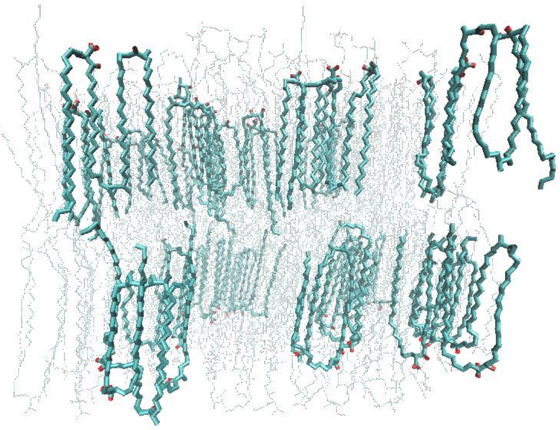

300 ns

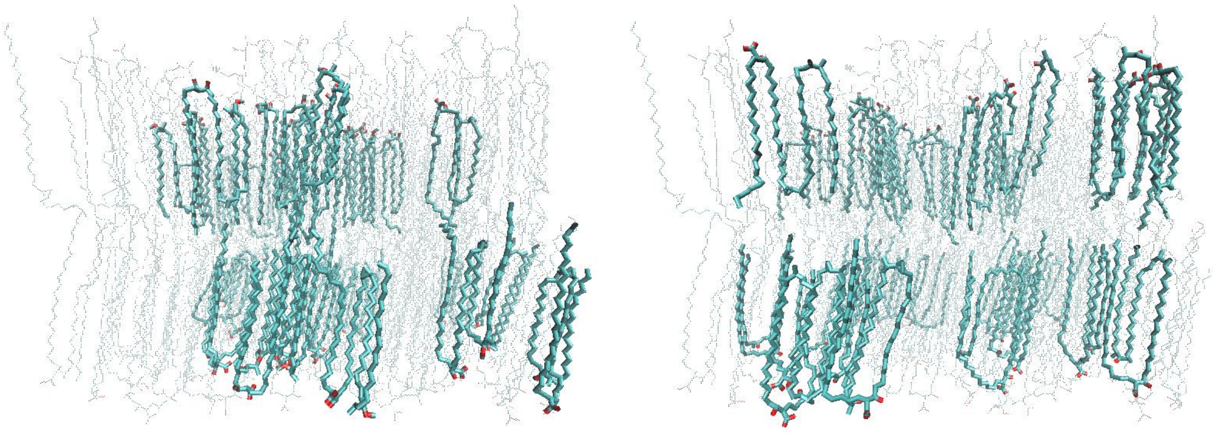

**Figure S7.** Conformations of MMA and KMA in a mixed membrane 140AMA(eU):30MMA:30KMA for the initial configuration (0), 100, 200 and 300 ns of molecular dynamics simulation. Weak halftone background represents the AMA molecules with eU conformation. The observation point passes through the XOY plane.
